# Digital Health Interventions for Hypertension Management in US Populations Experiencing Health Disparities: A Systematic Review and Meta-Analysis

**DOI:** 10.1001/jamanetworkopen.2023.56070

**Published:** 2024-02-14

**Authors:** Miriam E. Katz, Reed Mszar, Alyssa A. Grimshaw, Craig G. Gunderson, Oyere K. Onuma, Yuan Lu, Erica S. Spatz

**Affiliations:** 1 New York Medical College School of Medicine, Valhalla; 2 Department of Chronic Disease Epidemiology, Yale School of Public Health, New Haven, Connecticut; 3 Harvey Cushing/John Hay Whitney Medical Library, Yale University, New Haven, Connecticut; 4 Department of Medicine, Yale School of Medicine, New Haven, Connecticut; 5 VA Connecticut Healthcare System, West Haven; 6 Division of Cardiology, Massachusetts General Hospital, Boston; 7 Section of Cardiovascular Medicine, Yale School of Medicine, New Haven, Connecticut; 8 Yale Center for Outcomes Research and Evaluation, Yale New Haven Health, New Haven, Connecticut

## Abstract

**Importance:**

Hypertension remains a leading factor associated with cardiovascular disease, and demographic and socioeconomic disparities in blood pressure (BP) control persist. While advances in digital health technologies have increased individuals’ access to care for hypertension, few studies have analyzed the use of digital health interventions in vulnerable populations.

**Objective:**

To assess the association between digital health interventions and changes in BP and to characterize tailored strategies for populations experiencing health disparities.

**Data Sources:**

In this systematic review and meta-analysis, a systematic search identified studies evaluating digital health interventions for BP management in the Cochrane Library, Ovid Embase, Google Scholar, Ovid MEDLINE, PubMed, Scopus, and Web of Science databases from inception until October 30, 2023.

**Study Selection:**

Included studies were randomized clinical trials or cohort studies that investigated digital health interventions for managing hypertension in adults; presented change in systolic BP (SBP) or baseline and follow-up SBP levels; and emphasized social determinants of health and/or health disparities, including a focus on marginalized populations that have historically been underserved or digital health interventions that were culturally or linguistically tailored to a population with health disparities. The study followed the Preferred Reporting Items for Systematic Reviews and Meta-Analyses guideline.

**Data Extraction and Synthesis:**

Two reviewers extracted and verified data. Mean differences in BP between treatment and control groups were analyzed using a random-effects model.

**Main Outcomes and Measures:**

Primary outcomes included mean differences (95% CIs) in SBP and diastolic BP (DBP) from baseline to 6 and 12 months of follow-up between digital health intervention and control groups. Shorter- and longer-term follow-up durations were also assessed, and sensitivity analyses accounted for baseline BP levels.

**Results:**

A total of 28 studies (representing 8257 participants) were included (overall mean participant age, 57.4 years [range, 46-71 years]; 4962 [60.1%], female). Most studies examined multicomponent digital health interventions incorporating remote BP monitoring (18 [64.3%]), community health workers or skilled nurses (13 [46.4%]), and/or cultural tailoring (21 [75.0%]). Sociodemographic characteristics were similar between intervention and control groups. Between the intervention and control groups, there were statistically significant mean differences in SBP at 6 months (−4.24 mm Hg; 95% CI, −7.33 to −1.14 mm Hg; *P* = .01) and SBP changes at 12 months (−4.30 mm Hg; 95% CI, −8.38 to −0.23 mm Hg; *P* = .04). Few studies (4 [14.3%]) reported BP changes and hypertension control beyond 1 year.

**Conclusions and Relevance:**

In this systematic review and meta-analysis of digital health interventions for hypertension management in populations experiencing health disparities, BP reductions were greater in the intervention groups compared with the standard care groups. The findings suggest that tailored initiatives that leverage digital health may have the potential to advance equity in hypertension outcomes.

## Key Points

**Question:**

Are digital health interventions associated with reducing blood pressure (BP) levels in US populations experiencing health disparities, and what types of tailored modalities have been used to manage hypertension in demographically and socioeconomically diverse subgroups?

**Findings:**

In this systematic review and meta-analysis of 28 studies, patients with health disparities receiving a digital health intervention compared with standard care had greater reductions in systolic BP at 6 and 12 months. Most studies involved multicomponent interventions, primarily remote BP monitoring.

**Meaning:**

These findings suggest that digital health interventions are associated with improved BP levels in populations experiencing health disparities.

## Teaser

This systematic review and meta-analysis characterizes digital health interventions for reducing hypertension and assesses the association of these interventions with blood pressure changes in adults experiencing health disparities.

## Introduction

Hypertension is an important risk factor for cardiovascular disease (CVD), which accounts for approximately 1 in 5 deaths in the US.^1^ Nearly half (47%) of all US adults, or 116 million individuals, have hypertension (defined as systolic blood pressure [SBP]≥130 mm Hg or diastolic blood pressure [DBP]≥80 mm Hg). Only 24% of those with hypertension have their condition under control.^2,3^ Racial, ethnic, and socioeconomic disparities in hypertension prevalence, awareness, and treatment persist. Notably, population-based studies have shown that Black and Hispanic adults have lower BP awareness and control despite a higher overall burden of hypertension compared with non-Hispanic White individuals.^4,5,6,7,8^

Emerging research has shown digital health technology to be a promising avenue for managing uncontrolled hypertension, particularly in underserved populations impacted by barriers to accessing care. Current digital health approaches for hypertension management typically involve text message reminders for medication adherence,^9,10,11^ remote BP monitoring,^12,13^ and virtual behavioral coaching.^14,15^ There is also growing evidence for the value of tailored, multicomponent approaches for hypertension management.^16,17^ Home monitoring can identify “white-coat hypertension” (BP measurements are high in the clinic but normal at home) and masked hypertension (BP measurements are normal in the clinic but high at home) and empower patients to take more control over their health.^12,18^ When combined with a centralized medical team to respond to elevated home BP readings, home monitoring has potential to significantly improve BP control. Despite the intended benefits of home monitoring and other digital health interventions for hypertension control, there are varying effects on cardiovascular risk factor control, potentially because of the need for technology support and remote engagement.^19,20,21,22^ Studies that incorporate a social determinants of health framework in the development and implementation of digital health interventions could prevent further widening of the digital divide and existing health disparities.^23^

Accordingly, this systematic review and meta-analysis aimed to assess the association between digital health interventions and BP changes among populations experiencing health disparities. It also aimed to characterize the diversity of contemporary strategies used to meet the needs of populations experiencing health disparities.

## Methods

This systematic review and meta-analysis was conducted in accordance with the Preferred Reporting Items for Systematic Reviews and Meta-Analyses (PRISMA) reporting guideline.^24^ The study protocol and methods were registered with PROSPERO a priori (CRD42021257529).

### Search Strategy and Study Selection

A systematic search of the literature was conducted by a medical librarian (A.A.G.) in the Cochrane Library, Ovid Embase, Google Scholar, Ovid MEDLINE, PubMed, Scopus, and Web of Science Core Collection databases to identify relevant articles published from the earliest record in the respective database to October 30, 2023. Databases were searched using a combination of controlled vocabulary and free-text terms for digital health, hypertension, social determinants of health, and demographic and/or socioeconomic disparities. The search was not limited by publication type, language, or year. The search was peer reviewed by a second medical librarian using Peer Review of Electronic Search Strategies. Details of the full search strategy are listed in eTable 1 in Supplement 1. CitationChaser was used to search the reference lists of included studies and to retrieve articles that had cited the included studies to find additional relevant studies not retrieved by the database search.

Included studies were randomized clinical trials (RCTs) or cohort studies that investigated digital health interventions for managing hypertension and were conducted in adult populations (age ≥18 years). Studies were not excluded based on the type of digital health intervention used. Included studies presented change in SBP and/or baseline and follow-up SBP levels as primary or secondary outcomes. Studies were excluded if they possessed the following characteristics: review articles, abstracts, editorials or letters, animal studies, or case reports. Conference abstracts were excluded given that detailed information was required on follow-up BP levels, participants’ sociodemographic characteristics, and an in-depth description of the digital health intervention being assessed.

Additionally, given the objective of this systematic review and meta-analysis to assess the outcomes of digital health interventions in populations experiencing health disparities, the studies that were included at the full-text review stage were required to possess any of the following characteristics: (1) a clear emphasis on social determinants of health and/or health disparities, (2) study eligibility criteria focusing on the exclusive or predominant recruitment and enrollment of marginalized populations that have historically been underserved and underrepresented in medical and public health research, (3) study design and conduct approaches involving intentional community partnership and stakeholder engagement, and (4) digital health intervention strategies that were culturally and/or linguistically tailored to the populations they were meant to serve. It was not feasible to include these criteria as part of the first stage of eligibility determination because this information is frequently not included in a study’s title or abstract, thereby necessitating full review of the methods and results.

Citations from all databases were imported into an EndNote 20 library (Clarivate Analytics). Duplicate citations were removed using the Yale Reference Deduplicator. The deduplicated results were imported into Covidence for screening and data extraction. Two independent screeners (M.E.K., R.M.) performed a title and abstract review, and a third screener (E.S.S.) resolved disagreements. The full texts of the resulting studies were then reviewed for inclusion by 2 independent screeners (M.E.K., R.M.), with a third screener (E.S.S.) resolving disagreements.

### Statistical Analysis

#### Data Extraction and Analysis

Data were extracted and verified by 2 authors (M.E.K., R.M.). These data consisted of study characteristics including the following: study design (RCTs or cohort studies), study type (eg, pilot study status), study duration and location, type of digital health intervention(s), population characteristics and eligibility criteria, primary and secondary outcome measures, and type or level of cultural tailoring and community engagement. Additionally, we reported means and SDs or frequencies and proportions for the following sociodemographic characteristics for each study: age, sex, race and ethnicity (categories included non-Hispanic Black, non-Hispanic White, Hispanic, and other race [Asian and multiracial]), income, level of completed education, and insurance status and type. The outcomes of interest included baseline and follow-up SBP and DBP levels (in mm Hg) at 3, 6, 12, 18, or 24 months and SBP and DBP changes from baseline. In studies that either did not report the SD value for the BP outcomes or reported IQR or SE values instead, we used several algebraic conversions to produce the proper SD measure of variation to integrate into our meta-analysis.^25^

The methods of the meta-analysis were established prior to data extraction. Mean differences in BP between treatment and control groups were analyzed with random-effects meta-analysis using the restricted maximum likelihood method. Analysis was stratified by follow-up duration in months. Study heterogeneity was evaluated using Higgins *I^2^* statistics with thresholds of 25%, 50%, and 75%, corresponding with low, moderate, and high levels of heterogeneity, respectively.^25^ If the *I*^2^ value was 50% or greater, we explored heterogeneity using leave-one-out sensitivity analysis, subgroup analysis, and metaregression for outcomes with at least 10 studies. Subgroups included studies that tested remote BP monitoring in the intervention arm, focused on Black or Hispanic individuals, were pilot studies, identified BP as the primary outcome, and were limited to patients with controlled BP at baseline. Metaregression included the same subgroup variables and the proportion of study participants who were female, were Black or Hispanic, and/or had a lower level of completed education. Statistical analysis was performed using Stata/BE, version 17.0 (StataCorp LLC). Two-sided *P* < .05 was considered significant.

#### Assessment of Study Quality and Publication Bias

The quality of observational studies was assessed independently by 2 investigators (M.E.K., R.M.) and scored on the Newcastle-Ottawa Scale, and interobserver agreement was calculated using the Cohen *k* coefficient.^26^ Discrepancies were resolved by the senior reviewer (E.S.S.). Publication bias was assessed visually by inspection of a funnel plot and through the Egger test of intercept.^27^

## Results

### Study Characteristics

**Figure 1.**
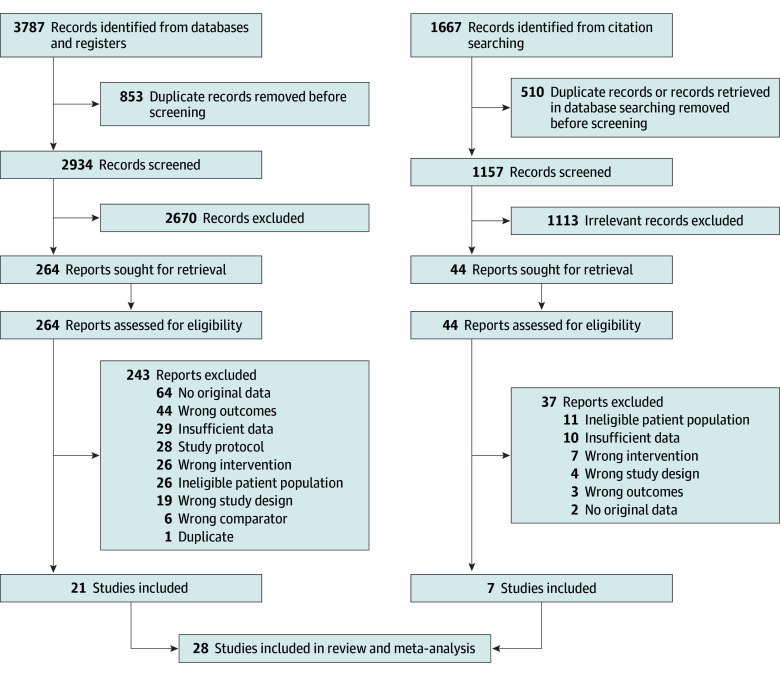
Flowchart Depicting Study Methods in Accordance With the Preferred Reporting Items for Systematic Reviews and Meta-Analyses Guideline

Our initial literature search yielded 4091 studies after removing duplicate publications (Figure 1). Of these, 308 full-text articles were evaluated, and 28 studies^28,29,30,31,32,33,34,35,36,37,38,39,40,41,42,43,44,45,46,47,48,49,50,51,52,53,54,55^ (27 RCTs [96.4%]^29,30,31,32,33,34,35,36,37,38,39,40,41,42,43,44,45,46,47,48,49,50,51,52,53,54,55^ and 1 cohort study [3.6%]^28^) were ultimately included in this systematic review and meta-analysis (Table 1 and Table 2). Among the included studies, the eligibility criteria for participant recruitment varied widely, although most used a diagnosis of hypertension and/or a history of taking antihypertensive medications (eTable 2 in Supplement 1).^28,30,31,32,33,36,37,41,42,43,44,46,47,48,49,50,51,52,53,54^ Eighteen studies (64.3%) included remote BP monitoring,^28,30,31,32,33,34,37,41,43,44,46,48,49,51,52,53,54,55^ and all studies incorporated multiple digital health components, including electronic health reminders, education, and behavioral support programs. Other interventions included the integration of community health workers (CHWs) or skilled nurses (13 studies [46.4%]^28,29,32,34,35,36,38,40,41,43,45,46,53^), wearable or ingestible sensors (4 studies [14.3%]^40,43,50,54^), and tailored messaging or reminders based on cultural, linguistic, behavioral, and/or psychosocial considerations (21 studies [75.0%]^29,31,32,33,34,35,36,38,39,40,41,42,43,45,46,47,48,52,53,54,55^) (eTable 3 in Supplement 1). Additionally, 5 studies (17.9%) directly involved active medication management for hypertension control as part of the digital health intervention.^28,35,41,43,48^ Studies excluded from this systematic review and meta-analysis and the corresponding rationales are presented in eTables 4 and 5 in Supplement 1. Few studies (4 [14.3%]) reported the BP outcomes of interest beyond 1 year of follow-up.

**Table 1.** Study Characteristics and Participant Information for the 28 Included Studies Meeting Eligibility Criteria

Author, year^a^	Pilot study	Study population	Focus on disparities	Follow-up, mo	Remote monitoring	Study location
Naqvi et al,^41^ 2022	Yes	Individuals with an acute ischemic or hemorrhagic stroke discharged after hospitalization with HTN	Involved a clinical site primarily serving a Hispanic, low-income community	3	Yes	Northern Manhattan, New York
Brewer et al,^40^ 2022	Yes	Black or African American individuals able to engage in moderate physical activity	Focused on Black or African American individuals	6	No	Rochester and Minneapolis–St Paul, Minnesota
Clark et al,^28^ 2021	No	Individuals with uncontrolled HTN	Focused on a predominately rural and low-income population	6	Yes	Mississippi
Schoenthaler et al,^42^ 2020	Yes	Black or African American patients with uncontrolled HTN and/or diabetes	Focused on Black or African American individuals	3	No	New York, New York
Vaughan et al,^35^ 2021	No	Hispanic, Spanish-speaking individuals with diabetes without insurance earning ≤250% of the federal poverty level	Focused on Hispanic individuals with a low income and no health insurance	6	No	Houston, Texas
Zha et al,^43^ 2020	Yes	Individuals with uncontrolled HTN taking anti-HTN medications and living in public housing units	Focused on individuals living in public housing units	6	Yes	Newark, New Jersey
Schroeder et al,^31^ 2020	No	Individuals with HTN	Focused on multiple racial and ethnic groups receiving care at an Urban Indian Health Organization	12	Yes	Albuquerque, New Mexico
Still et al,^32^ 2020	Yes	African American individuals with HTN prescribed anti-HTN medications	Focused on Black or African American individuals	3	Yes	Cleveland, Ohio
Persell et al,^44^ 2020	No	Individuals with HTN	Included a large proportion of Black or African American individuals	6	Yes	Chicago, Illinois
Tuot et al,^45^ 2019	Yes	Individuals with CKD with recent clinical visit(s)	Focused on individuals with low income receiving care at safety-net clinics; intervention was language concordant and culturally tailored	12	No	San Francisco, California
Chandler et al,^46^ 2019	No	Hispanic individuals with HTN and prescribed anti-HTN medications	Focused on Hispanic individuals	9	Yes	Charleston County, South Carolina
Bennett et al,^47^ 2018	No	Individuals with HTN, obesity, diabetes, and hyperlipidemia	Focused on socioeconomically disadvantaged primary care patients	12	No	Central North Carolina
Bosworth et al,^48^ 2018	No	Veterans enrolled at 1 of 3 primary care clinics with HTN or hypercholesterolemia	Focused on US veterans	12	Yes	North Carolina and Virginia
Skolarus et al,^33^ 2018	Yes	Individuals with HTN	Used a community-based participatory research framework and focused on Black or African American individuals	6	Yes	Flint, Michigan
Morawski et al,^49^ 2018	No	Individuals with HTN on anti-HTN medications	Included a large proportion of Black or African American individuals	3	Yes	Not indicated or unclear
Fortmann et al,^29^ 2017	No	Uninsured or underinsured Hispanic individuals with diabetes	Focused on Hispanic individuals with minimal or no health insurance	6	No	San Diego and Riverside, California
Frias et al,^50^ 2017	Yes	Individuals with uncontrolled HTN and diabetes with previously failed HTN treatment	Included a large proportion of Hispanic individuals and those with low income	3	No	California and Colorado
Bove et al,^37^ 2013	No	Individuals with HTN	Conducted in an underserved, urban community and included a large proportion of Black or African American individuals	6	Yes	Philadelphia, Pennsylvania, and Wilmington, Delaware
Crowley et al,^34^ 2013	No	Black individuals with diabetes with recent clinical visit(s)	Focused on Black or African American individuals	12	Yes	Durham, North Carolina
Rifkin et al,^51^ 2013	No	Individuals with stage 3 CKD and established HTN attending a VA clinic	Conducted among older veterans	6	Yes	San Diego, California
Margolis et al,^30^ 2013	No	Individuals with uncontrolled HTN	Included a large proportion of individuals with low income	12	Yes	Minneapolis–St Paul, Minnesota
Bennett et al,^36^ 2012	No	Individuals with a BMI of 30-50, weighing <180 kg, taking anti-HTN medications, and with recent clinical visit(s)	Focused on socioeconomically disadvantaged individuals with a large proportion of Black or African American individuals	24	No	Boston, Massachusetts
McKee et al,^52^ 2011	Yes	Individuals with HTN receiving care for diabetes	Tailored intervention to a multiethnic, low-income, primary care population	6	Yes	Bronx, New York
Frosch et al,^38^ 2011	No	Individuals with diabetes with recent clinical visit(s)	Focused on low-income, uninsured, and ethnically diverse patients	6	No	Los Angeles, California
Anderson et al,^39^ 2010	No	Individuals with diabetes with recent clinical visit(s)	Tailored intervention to meet the cultural and linguistic needs of an underserved, predominantly Hispanic population	12	No	Connecticut
Brennan et al,^53^ 2010	No	Black individuals with HTN and a PCP	Focused on Black or African American individuals	12	Yes	Not indicated or unclear
Bosworth et al,^54^ 2009	No	Individuals with HTN taking anti-HTN medications and residing in specific zip codes with an upcoming PCP appointment	Tailored intervention to patients’ literacy and social support among a large proportion of Black or African American individuals	24	Yes	North Carolina
Shea et al,^55^ 2009	No	Medicare beneficiaries with diabetes	Focused on Medicare beneficiaries living in a medically underserved area	12	Yes	New York

Abbreviations: BMI, body mass index (calculated as weight in kilograms divided by height in meters squared); CKD, chronic kidney disease; HTN, hypertension; PCP, primary care physician; VA, US Department of Veterans Affairs.

^a^ All included studies were randomized clinical trials except for Clark et al,^28^ which was a prospective cohort study.

**Table 2.** Specific Information on the Digital Health Intervention Components Used for the 28 Included Studies Meeting the Eligibility Criteria

Study	Digital health intervention component
Naqvi et al,^41^ 2022	Home BP monitoring devices with wireless transmission and electronic tablet devices Nurses telephoned patients for severely elevated BP (>180/110 mm Hg), assessed for concerning symptoms, notified the physician who would decide whether additional intervention was needed by team members, and reinforced changes to antihypertensive medications
Brewer et al,^40^ 2022	FAITH! app with features including participant dashboard with tailored messaging, diet and physical activity goal chart, cardiovascular health education modules, and interactive self-monitoring of fruit and vegetable intake and physical activity Discussion platform for participant interaction of healthy lifestyle practices, church leadership and previous FAITH! program participant video accounts on personal experiences, and cookbooks including heart-healthy traditional African American cuisine
Clark et al,^28^ 2021	Telemonitoring kit including electronic tablet and home BP monitor
Schoenthaler et al,^42^ 2020	Tailoring survey based on the information-motivation-behavioral adherence questionnaire and individualized adherence profile Personalized list of interactive adherence-promoting modules that were matched to the barriers outlined on the adherence profile Culturally tailored modules including narratives by Black patients that discuss importance of taking medications in the context of life values and positive voice videos that allow patients to hear about other Black patients’ experiences with hypertension and diabetes
Vaughan et al,^35^ 2020	CHW-led diabetes group visits (large group education and small group addressing medical, social, and behavioral barriers to care) To address challenges in low-income settings, the bilingual study physician prescribed medications offered at a low cost
Zha et al,^43^ 2020	Wireless BP wrist monitor with paired, free mobile application
Schroeder et al,^31^ 2020	Interactive voice-response and text-messaging reminders and weekly motivational messages to encourage healthy behaviors (in English or Spanish); home BP monitor Messages were culturally tailored and reviewed by an American Indian psychologist and the FNCH Advisory Council
Still et al,^32^ 2020	Web-based education modules; self–BP monitoring; free medication management app that provided SMS reminders and education to enhance medication adherence Nurse counseling from the Cleveland Council of Black Nurses, who provided culturally appropriate education materials
Persell et al,^44^ 2020	Wireless BP monitor with conversational artificial intelligence smartphone app using cognitive behavioral therapy
Tuot et al,^45^ 2019	The patient intervention was a comprehensive CKD-SMS program based on constructs of social cognitive theory: behavioral capability, self-efficacy, expectations, and reinforcement; the program was delivered by 2 full-time bilingual health coaches The program had 3 distinct elements: (1) language-concordant, low-literacy written patient educational materials; (2) a language concordant and culturally tailored automated telephone self-management program that reviewed kidney health topics; and (3) telephone-based health coaching delivered by lay bilingual health coaches trained in motivational interviewing and action planning
Chandler et al,^46^ 2019	Smart phone application with paired Bluetooth BP monitor Tailored motivational and social reinforcement messages Study design input from Hispanic clinical research staff and Hispanic adults with hypertension
Bennett et al,^47^ 2018	App-based BP self-monitoring with tailored feedback and a smart scale Dietitian-delivered counseling calls
Bosworth et al,^48^ 2018	Home BP monitor; clinical-pharmacist behavioral and telemedicine intervention to promote healthy behaviors using motivational interviewing
Skolarus et al,^33^ 2018	BP self-monitoring; tailored behavioral text messages Messages were culturally relevant to African American individuals and provided geographically relevant options for physical activity
Morawski et al,^49^ 2018	Medication adherence application that sends reminders and allows BP tracking with cuff
Fortmann et al,^29^ 2017	Received diabetes educational video, blood glucose meter, and testing strips Bilingual research assistants; text messages derived from culturally appropriate diabetes self-management education program; ongoing support via motivational messages
Frias et al,^50^ 2017	Digital medicine offering consisting of an ingestible sensor (inside a placebo pill), an adhesive wearable sensor patch, patient mobile app, and provider web portal
Bove et al,^37^ 2013	Home BP monitor, scale, and a pedometer; BP education
Crowley et al,^34^ 2013	Self-management education delivered by nurse interventionists with training in motivational interviewing Intervention materials designed for patients with low income or low literacy Research staff underwent interactive training on cultural sensitivity and awareness of issues facing African American individuals in the community
Rifkin et al,^51^ 2013	Fully automatic BP unit and the home health hub
Margolis et al,^30^ 2013	Home BP monitor; pharmacist-led education on healthy behaviors
Bennett et al,^36^ 2012	Behavioral weight loss intervention designed for use in resource-constrained settings, including populations with limited literacy (monitoring options in English and Spanish) Community health educators delivered counseling calls, were trained in motivational interviewing, and led optional group sessions Provided tailored information on community resources that encouraged healthy behaviors
McKee et al,^52^ 2011	Home health nurses with training in self-management and healthy-behavior counseling
Frosch et al,^38^ 2011	Educational materials and multiple sessions of telephone coaching with bilingual nurse educators trained in patient-centered approaches to diabetes management
Anderson et al,^39^ 2010	Tailored telephonic disease management with nurses on healthy behaviors Educational materials were in English and Spanish and at a 4th-grade reading level
Brennan et al,^53^ 2010	BP monitors; nurses providing culturally competent disease management Educational materials developed for African American individuals based on well-established guides
Bosworth et al,^54^ 2009	Tailored behavior self-management intervention; home BP monitor Nurse-led telephone calls on healthy behaviors with favorable readability score (<9th-grade reading level)
Shea et al,^55^ 2009	Home telemedicine unit including web camera for video conferencing with nurse case managers and home glucose meter with a blood pressure cuff Educational web page in English in Spanish and in regular and low-literacy versions

Abbreviations: BP, blood pressure; CHW, community health worker; CKD, chronic kidney disease; FAITH!, Fostering African American Improvement in Total Health; FNCH, First Nations Community Healthsource; SMS, short message service.

### Baseline Sociodemographic Characteristics

Overall, 8257 individuals from the 28 studies were included in this systematic review and meta-analysis, of whom 3828 (46.4%) were assigned to an intervention group and 4429 (53.6%) were assigned to a control group. The mean pooled age of participants was 57.4 years (range, 46-71 years); 3295 (39.9%) were men, and 4962 (60.1%) were women (eTable 3 in Supplement 1). Overall, 1631 individuals (19.8%) were Hispanic; 3531 (42.8%), non-Hispanic Black; 2607 (31.6%), non-Hispanic White; and 488 (5.9%), other race. Regarding socioeconomic characteristics, 1471 individuals (17.8%) had a low level of completed education (often defined as less than high school), and 1884 (22.8%) were classified as having low income. Additionally, 1177 individuals (14.3%) had Medicaid, 2146 (26.0%) had Medicare, and 548 (6.6%) had no insurance coverage. In total, 17 studies (60.7%) focused on Black or Hispanic individuals or included a large proportion of individuals self-identifying as Black or Hispanic.^29,32,33,34,35,36,37,39,40,41,42,44,46,49,50,53,54^ Regarding socioeconomic characteristics, 15 studies (53.6%) were specifically conducted in socioeconomically disadvantaged communities or among individuals with a low income or who were uninsured or underinsured.^28,29,30,35,36,37,38,39,41,43,45,47,50,52,55^ Several studies were also conducted among veterans (2 [7.1%]^48,51^) and rural populations (3 [10.7%]^28,47,54^).

### SBP Changes

Across all studies included in the meta-analysis regardless of follow-up duration, the mean (SD) SBP at baseline for the digital health intervention and control groups was 138.6 (16.3) mm Hg and 139.2 (16.2) mm Hg, respectively. The mean (SD) follow-up SBP at the final follow-up time point was 131.8 (15.9) mm Hg in the intervention groups and 135.3 (16.5) mm Hg in the control groups.

**Figure 2.**
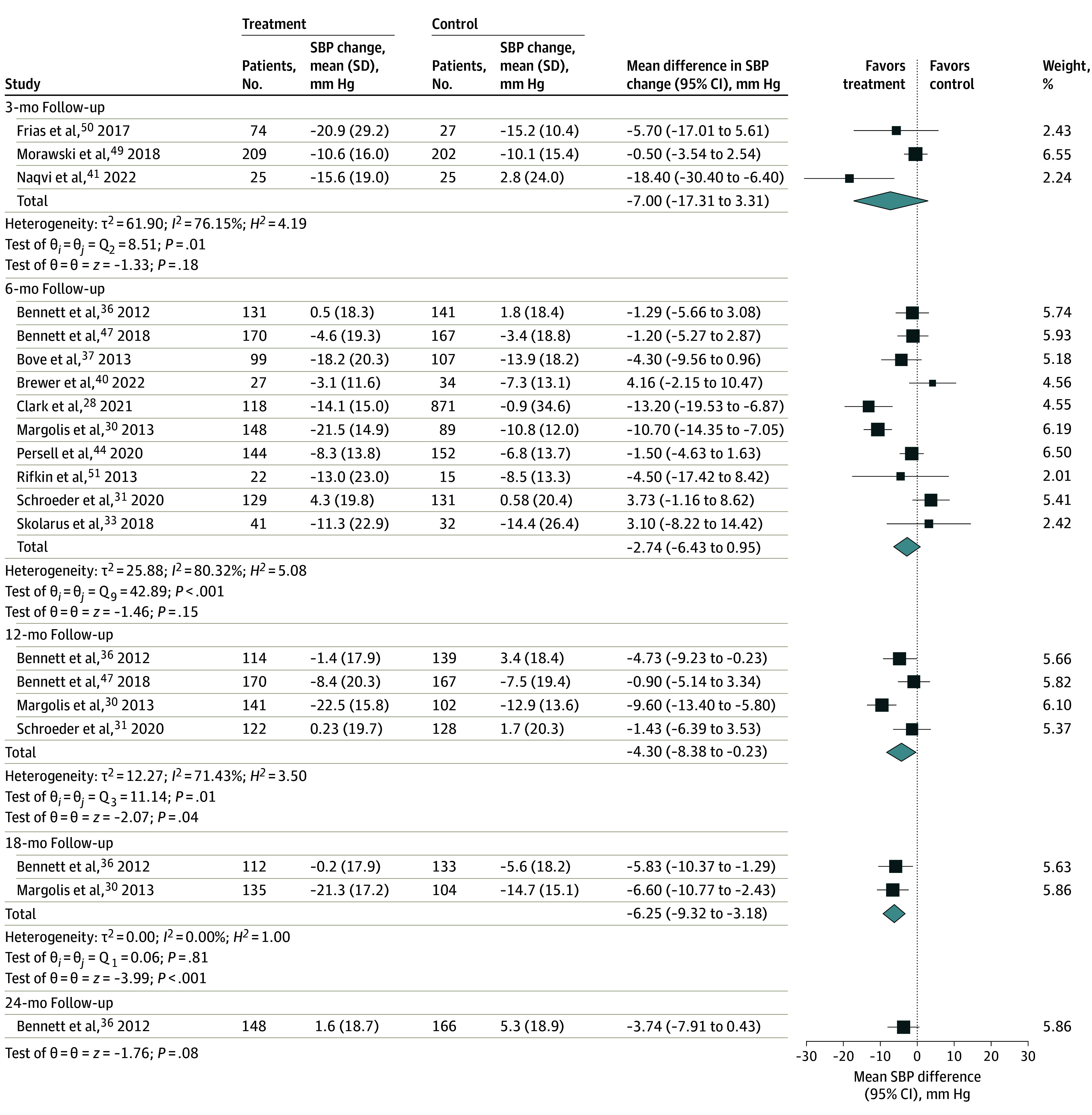
Differences in Systolic Blood Pressure (SBP) Changes From Baseline to Follow-Up Time Points Between Digital Health Intervention and Control Groups Random-effects restricted maximum likelihood model. Squares indicate mean SBPs, with horizontal lines indicating 95% CIs and the size of the squares representing weight; diamonds indicate pooled estimates, with outer points of the diamonds indicating 95% CIs.

**Figure 3.**
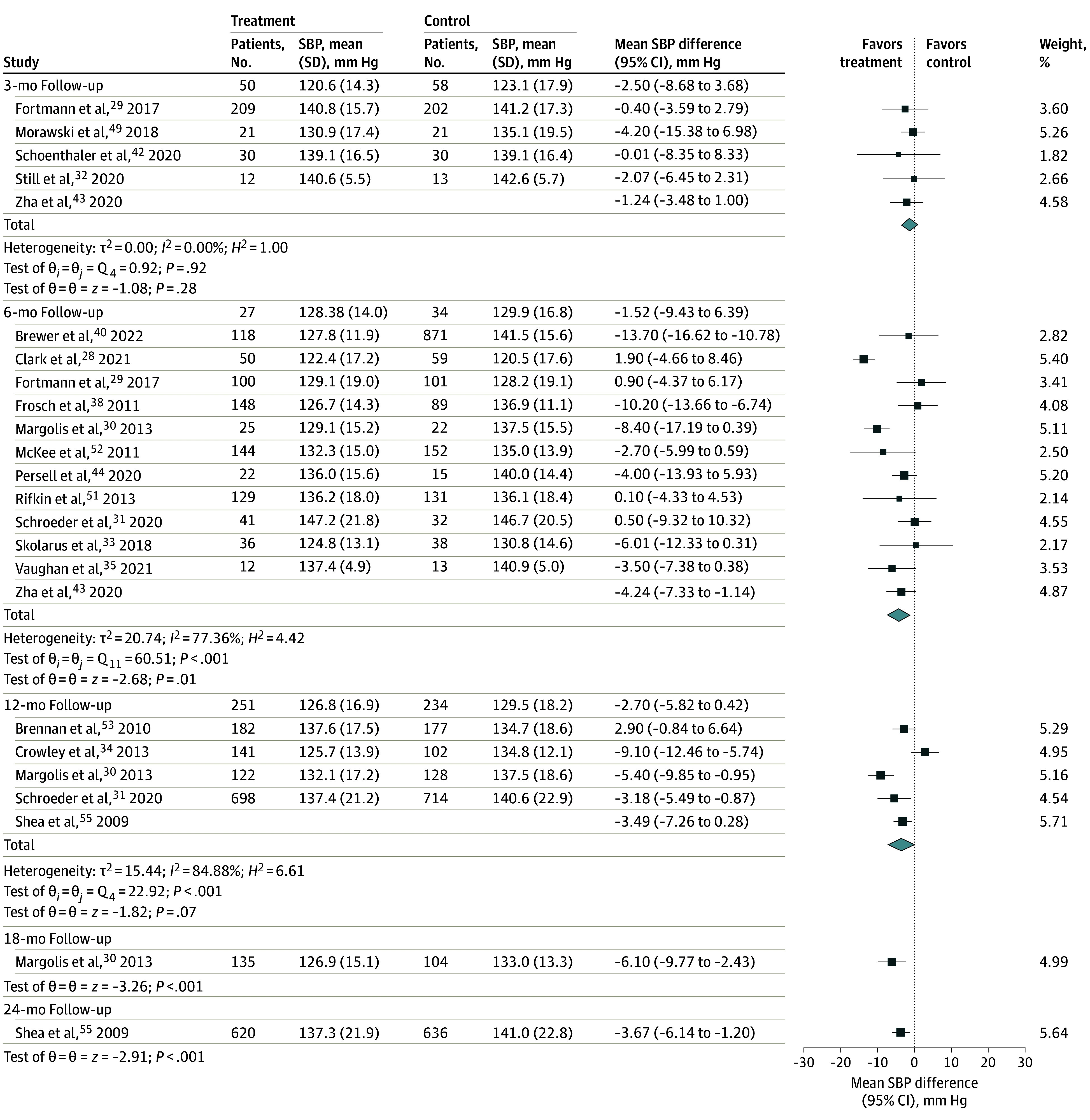
Differences in Follow-Up Systolic Blood Pressure (SBP) Between Digital Health Intervention and Control Groups at Different Time Points Random-effects restricted maximum likelihood model. Squares indicate mean SBPs, with horizontal lines indicating 95% CIs and the size of the squares representing weight; diamonds indicate pooled estimates, with outer points of the diamonds indicating 95% CIs.

Among the 10 studies that reported change in SBP at 6 months,^28,30,31,33,36,37,40,44,47,51^ we found a mean difference of −2.74 mm Hg (95% CI, −6.43 to 0.95 mm Hg; *P* = .15; *I^2^* = 80.32%) between the digital health intervention and control groups (Figure 2). At 12 months’ follow-up, there was a statistically significant mean difference of −4.30 mm Hg (95% CI, −8.38 to −0.23 mm Hg; *P* = .04; *I^2^* = 71.43%) in the 4 studies reporting SBP change.^30,31,36,47^ For the SBP change outcome, 3 or fewer studies had complete information at the 3-month,^41,49,50^ 18-month,^30,36^ and 24-month^36^ follow-up. Among the 12 studies that presented follow-up SBP, we found a statistically significant mean difference of −4.24 mm Hg (95% CI, −7.33 to −1.14 mm Hg; *P* = .01; *I^2^* = 77.36%) at 6 months (Figure 3).^28,29,30,31,33,35,38,40,43,44,51,52^ The greatest mean difference in 6-month SBP between intervention and control groups was −13.70 mm Hg (95% CI, −16.62 to −10.78 mm Hg),^28^ while the smallest (most positive) mean difference was 1.90 mm Hg (95% CI, −4.66 to 8.46 mm Hg).^29^

### DBP Changes

Across all included studies regardless of follow-up duration, the mean (SD) DBP at baseline for the digital health intervention and control groups was 81.4 (11.8) mm Hg and 81.7 (11.7) mm Hg, respectively. The mean follow-up DBP at the end of the included studies (time of last follow-up) was 77.8 (10.5) mm Hg in the intervention groups and 79.6 (11.2) mm Hg in the control groups.

Among 10 studies that reported 6-month DBP changes, we found a mean difference of −1.11 mm Hg (95% CI, −3.09 to 0.87 mm Hg; *P* = .27; *I^2^* = 70.45%) (eFigure 1 in Supplement 1).^28,30,31,33,36,37,40,44,47,51^ The most prominent mean difference in DBP changes between the intervention and control groups was −6.10 mm Hg (95% CI, −11.02 to −1.18 mm Hg).^28^ Across the 12 studies with 6-month data on follow-up DBP (42.9%), we found a mean difference of −1.86 mm Hg (95% CI, −3.82 to 0.10 mm Hg; *P* = .06; *I^2^* = 67.57%) (eFigure 2 in Supplement 1).^28,29,30,31,33,35,38,40,43,44,51,52^ The largest mean difference was −6.70 mm Hg (95% CI, −9.98 to −3.42 mm Hg),^30^ while the smallest (most positive) mean difference between the intervention and control groups was 1.50 mm Hg (95% CI, −1.53 to 4.53 mm Hg).^31^

### Subgroup Analysis, Metaregression, and Sensitivity Analysis

Among the studies with 6-month follow-up data for the BP outcomes of interest,^28,29,30,31,33,35,38,40,43,44,51,52^ each of the 6-month outcomes had moderate-high heterogeneity (*I*^2^ = 67.57%-80.32%). On sensitivity analysis, removing individual studies had minimal effect on the pooled result (eFigures 3-6 in Supplement 1). Subgroup analyses based on whether studies included remote BP monitoring, were limited to specific racial or ethnic groups, were pilot studies, enrolled patients with controlled BP at baseline, or had BP as the primary outcome also found little effect on statistical heterogeneity (eTables 5-9 in Supplement 1). Metaregression using the proportion of study participants who were female, Black, or Hispanic and/or had a lower level of education had little effect on heterogeneity. Similarly, metaregression including the subgroup variables had little effect on heterogeneity except whether the mean baseline BP was controlled (SBP ≤140 mm Hg). For the outcome of change in SBP, 4 studies had a mean baseline SBP of 140 mm Hg or lower.^31,36,40,47^ On metaregression, 50.3% of the heterogeneity was explained by this variable (β = 7.1; *P* = .02). For the other 3 BP outcomes at 6 months, the studies with a mean baseline SBP of 140 mm Hg or lower^29,31,35,36,38,40,47,52^ found less benefit of digital health interventions, but the differences were not statistically significant.

### Publication Bias

Among included studies presenting complete outcome information such as change in BP or follow-up BP,^28,29,30,31,32,33,34,35,36,37,38,40,41,42,43,44,47,49,50,51,52,53,55^ inclusion of fewer than 10 studies limited formal assessment of publication bias (eFigures 3-6 in Supplement 1). Given that only the 6-month time point had 10 or more studies reporting BP changes or BP at follow-up, this was the only duration that we were able to assess for publication bias. The Egger regression intercept showed no significant publication bias among studies that reported change in SBP,^28,30,31,33,36,37,40,44,47,51^ follow-up SBP,^28,29,30,31,33,35,38,40,43,44,51,52^ change in DBP,^28,30,31,33,36,37,40,44,47,51^ and follow-up DBP.^28,29,30,31,33,35,38,40,43,44,51,52^

### Study Quality

Overall, there were few concerns regarding the quality of included studies in our systematic review and meta-analysis. The domain-specific judgements on study quality ranged from low risk of bias to some concerns of potential bias. In total, 8 of the 28 studies (28.6%) had some concerns, most often attributed to bias due to missing outcome data (eFigure 7 in Supplement 1).^28,33,34,40,41,45,50,52^ Only 1 study (3.6%) had an overall judgement score of “some concerns.”^34^

## Discussion

To our knowledge, this was the first large-scale, contemporary analysis of more than 8257 participants from 28 studies to characterize existing evidence on the outcomes of digital health interventions for hypertension management in populations experiencing health disparities. Our systematic review and meta-analysis included a diverse sample of participants and a breadth of culturally tailored strategies seeking to integrate racial, ethnic, and socioeconomic determinants into the study design and intervention delivery. We found statistically significant and clinically relevant mean differences in SBP at 6 months (−4.24 mm Hg) and SBP changes at 12 months (−4.30 mm Hg). Only 3 studies^30,36,55^ assessed BP changes beyond 1 year of follow-up.

This study found evidence of BP improvements in populations experiencing health disparities, strengthening the case for digital health as an efficient and effective tool for hypertension management in these groups. At 6 months of follow-up, individuals who received a digital health intervention had a 4.24 mm Hg greater reduction in SBP compared with those in a control group. Importantly, these results are consistent with findings from meta-analyses that focused on digital health interventions to lower BP levels in the general population.^17,56,57,58^ One systematic review conducted among 4271 participants from 11 RCTs demonstrated net changes of −3.85 mm Hg in SBP and −2.19 mm Hg in DBP in the combined digital health intervention group,^56^ which are similar to the mean differences observed in the current study.

The findings of our study can be evaluated in the context of the growing body of evidence linking health disparities with hypertension management and CVD. While this systematic review and meta-analysis focused on populations experiencing health disparities and assessed digital health interventions for culturally tailored components, we found that 17 studies were specifically focused on enrolling a large proportion of Black and Hispanic individuals.^29,32,33,34,35,36,37,39,40,41,42,44,46,49,50,53,54^ However, subgroup analysis based on whether studies were limited to specific racial or ethnic groups had little effect on statistical heterogeneity. Black and Hispanic adults and individuals with low income and lower level of completed education experience a disproportionately higher burden of hypertension and have higher rates of morbidity and mortality associated with CVD.^2,59,60^ Moreover, individuals without insurance have been shown to have worse CVD outcomes.^60,61^ These inequities may be driven by individual-, relational-, and system-level inequities. For example, lifestyle behaviors (ie, physical activity, diet, and sleep quality), interpersonal and structural racism and implicit bias, and differences in access to high-quality care can impact BP control rates.^62^ Black and Hispanic individuals face an increased level of discrimination, which has been associated with hypertension.^63^ In recent years, Hispanic and non-Hispanic Black individuals in the US have shown a stagnation and even a decline in hypertension awareness, treatment, and control, with widening gaps in BP control.^5,6,64^

Several of the included studies addressed these challenges. For example, we observed a diverse range of recruitment strategies and culturally tailored interventions, ranging from faith-based community partnerships to motivational coaching based on personal belief frameworks. While nearly all of the studies included a combination of home BP monitoring with synchronized digital cuffs, medication adherence messaging, or motivational reminders, 21 studies made these reminders linguistically and/or culturally tailored to their patient population.^29,31,32,33,34,35,36,38,39,40,41,42,43,45,46,47,48,52,53,54,55^ In the Cholesterol, Hypertension, and Glucose Education (CHANGE) study focusing on non-Hispanic Black patients with diabetes, nurses underwent cultural sensitivity training that provided information on the unique challenges that non-Hispanic Black individuals face in their community.^34^ Additionally, there was a diverse set of community-engaged aspects of the study design and conduct, including the integration of community health centers, involvement of local church leadership for faith-based recruitment and intervention delivery, and participation of community health educators and patient advisory councils.^29,31,32,33,35,36,37,38,39^ Community-based interventions have been shown to ease the psychosocial stressors often associated with clinical settings, such as white coat syndrome, along with building trust between research staff and study participants.^17^

Given our aim to characterize approaches to tailoring digital health intervention delivery for populations experiencing health disparities, we have provided several examples to help inform future work seeking to expand access to these strategies. The Reach Out Churches study by Skolarus et al^33^ was conducted across community centers and places of worship. The community-based participatory research intervention was designed by community and academic leaders to address needs such as food insecurity, cost-related medication nonadherence, poverty, and health literacy in a majority Black neighborhood. Although BP reduction was not statistically significant in that pilot trial, high participation and engagement provided evidence for the feasibility of community-based programs to focus on high-risk groups that are otherwise difficult to reach via traditional medical avenues. Additionally, in the Fostering African American Improvement in Total Health (FAITH!) trial, Brewer and colleagues^40^ developed and analyzed a community-informed mobile health intervention (FAITH! app) for promoting ideal cardiovascular health among African American individuals in faith communities. In addition to organizing an advisory board composed of diverse community stakeholders to provide study oversight and ensure community centeredness, the research team convened joint congregation community recruitment kickoff events and developed educational materials incorporating practical strategies to overcome barriers from social determinants of health.

In recent years, research has shown that hypertension management and control are low across all Hispanic and Latino groups, with rates lower than those among non-Hispanic White individuals and lowest among Hispanic adults without health insurance.^65,66^ The integration of CHWs has been a well-studied and validated approach to increasing health care access in these groups. Previous evidence has shown that CHWs who provide technical support, engage in participant recruitment, and are knowledgeable about community resources collectively aid in improving the adoption and acceptability of a digital health intervention.^67^ In this systematic review and meta-analysis, a subset of studies specified whether an intervention was available in the participants’ native or preferred language and whether culturally sensitive messaging was present, both of which would further enhance access to digital health interventions. In particular, Still et al^32^ partnered with nurses from the Cleveland Council of Black Nurses, who, similar to CHWs, served as a bridge between underserved communities and their health care needs. While the intervention and control groups did not have significant differences in BP control at 3 months, clinically relevant BP reduction was observed in the intervention group. Additionally, in the TIME Study, Vaughan and colleagues^35^ incorporated CHW-participant mobile health communication, CHW-led diabetes group visits, and CHW-physician diabetes training and support via telehealth in a population of Hispanic and Spanish-speaking individuals. Compared with control participants, those enrolled in TIME had significant BP improvement (SBP: −6.89 mm Hg vs 0.03 mm Hg; DBP: −3.36 mm Hg vs 0.2 mm Hg).

### Limitations

The findings from our systematic review and meta-analysis should be interpreted in the context of several limitations. First, this study was limited in its ability to examine comprehensive, patient-level data on BP changes beyond 1 year. With few studies reporting longer-term follow-up data and the proportion of individuals with controlled hypertension at the end of the study period, the outcomes that were sufficiently powered both overall and for subgroup analyses were limited. However, despite this limitation, statistically significant and clinically meaningful data for SBP changes at 6 and 12 months were identified. Second, since there was inconsistently reported information on sociodemographic characteristics and studies used different definitions for specific subpopulations experiencing health disparities, our literature search may not have captured all studies of digital health interventions conducted in these populations. For example, studies conducted in rural areas may not have been identified if not cataloged as such. To improve our capture of studies, we used a snowball approach, identifying studies referenced by articles that did meet our search criteria.

Third, several studies involved significant investment in community partnerships, patient engagement, and digital health interventions. While questions related to cost, sustainability, and scalability were beyond the scope of this study, they remain important challenges that should be considered in future interventions to address disparities in hypertension control. Last, given that many of the digital health interventions possessed multiple components, we were unable to isolate the effects of each component. Future scoping reviews may be particularly helpful in assessing the breadth of and heterogeneity in digital health intervention components. Relatedly, the control or standard care groups varied widely across included studies and may have impacted the observed effects accordingly.

## Conclusions

In this systematic review and meta-analysis of digital health interventions for hypertension management in populations experiencing health disparities, significant and clinically relevant differences in BP lowering between the intervention and control groups were detected. We also identified a breadth of interventions and community engagement strategies, such as participant recruitment and educational programming through faith-based organizations and community centers; however, few studies were conducted beyond 1 year. With the increased use of digital health technologies in medicine, it is important that researchers, clinicians, and public health professionals continue to adapt digital health interventions to meet the needs of demographically and socioeconomically diverse populations with different challenges to improving BP control. More personalized approaches to remote BP monitoring may help to eliminate inequities in hypertension management and outcomes.

## Notes

Supplement 1. **eTable 1.** Search Strategies and Additional Methods **eTable 2.** Additional Study Characteristics for the 28 Included Studies Meeting Eligibility Criteria **eTable 3.** Baseline Demographic and Socioeconomic Characteristics of Participants in the 28 Included Studies **eTable 4.** Excluded Studies From the Original Search With Reasons for Exclusion **eTable 5.** Excluded Studies That Were Eliminated From the Citation Chasing With Corresponding Rationale **eTable 6.** Subgroup Analysis Showing Change in SBP at 6 Months **eTable 7.** Subgroup Analysis Showing Change in DBP at 6 Months **eTable 8.** Subgroup Analysis Showing SBP Values at 6 Months **eTable 9.** Subgroup Analysis Showing DBP Values at 6 Months **eFigure 1.** Differences in Diastolic Blood Pressure Changes From Baseline to Follow-Up Time Points Between Digital Health Intervention and Control Groups **eFigure 2.** Differences in Follow-Up Diastolic Blood Pressures Between Digital Health Intervention and Control Groups at Different Time Points **eFigure 3.** Sensitivity Analysis for Presence of Publication Bias Using Change in SBP Outcome at 6-Month Follow-Up and Leave-One-Out Meta-Analysis Results for Same Outcome **eFigure 4.** Sensitivity Analysis for Presence of Publication Bias Using Follow-Up SBP Value Outcome at 6-Month Follow-Up and Leave-One-Out Meta-Analysis Results for Same Outcome **eFigure 5.** Sensitivity Analysis for Presence of Publication Bias Using Change in DBP Outcome at 6-Month Follow-Up and Leave-One-Out Meta-Analysis Results for Same Outcome **eFigure 6.** Sensitivity Analysis for Presence of Publication Bias Using Follow-Up DBP Value Outcome at 6-Month Follow-Up and Leave-One-Out Meta-Analysis Results for Same Outcome **eFigure 7.** Risk of Bias Assessment for Included Studies

Supplement 2.
Data Sharing Statement
