# Supplementary material for: Digital Health Interventions for Hypertension Management in US Populations Experiencing Health Disparities: A Systematic Review and Meta-Analysis
Source: JAMA Netw Open. 2024 Feb 14;7(2):e2356070. doi: 10.1001/jamanetworkopen.2023.56070 (PMC10867699; doi:10.1001/jamanetworkopen.2023.56070)
Supplement: Supplement 1. — eTable 1. Search Strategies and Additional Methods eTable 2. Additional Study Characteristics for the 28 Included Studies Meeting Eligibility Criteria eTable 3. Baseline Demographic and Socioeconomic Characteristics of Participants in the 28 Included Studies eTable 4. Excluded Studies From the Original Search With Reasons for Exclusion eTable 5. Excluded Studies That Were Eliminated From the Citation Chasing With Corresponding Rationale eTable 6. Subgroup Analysis Showing Change in SBP at 6 Months eTable 7. Subgroup Analysis Showing Change in DBP at 6 Months eTable 8. Subgroup Analysis Showing SBP Values at 6 Months eTable 9. Subgroup Analysis Showing DBP Values at 6 Months eFigure 1. Differences in Diastolic Blood Pressure Changes From Baseline to Follow-Up Time Points Between Digital Health Intervention and Control Groups eFigure 2. Differences in Follow-Up Diastolic Blood Pressures Between Digital Health Intervention and Control Groups at Different Time Points eFigure 3. Sensitivity Analysis for Presence of Publication Bias Using Change in SBP Outcome at 6-Month Follow-Up and Leave-One-Out Meta-Analysis Results for Same Outcome eFigure 4. Sensitivity Analysis for Presence of Publication Bias Using Follow-Up SBP Value Outcome at 6-Month Follow-Up and Leave-One-Out Meta-Analysis Results for Same Outcome eFigure 5. Sensitivity Analysis for Presence of Publication Bias Using Change in DBP Outcome at 6-Month Follow-Up and Leave-One-Out Meta-Analysis Results for Same Outcome eFigure 6. Sensitivity Analysis for Presence of Publication Bias Using Follow-Up DBP Value Outcome at 6-Month Follow-Up and Leave-One-Out Meta-Analysis Results for Same Outcome eFigure 7. Risk of Bias Assessment for Included Studies [file jamanetwopen-e2356070-s001.pdf]

Supplementary Online Content

Katz ME, Mszar R, Grimshaw AA, et al. Digital health interventions for hypertension management in US populations experiencing health disparities: a systematic review and meta-analysis. *JAMA Netw Open*. 2024;7(2):e2356070. doi:10.1001/jamanetworkopen.2023.56070

eTable 1. Search Strategies and Additional Methods

eTable 2. Additional Study Characteristics for the 28 Included Studies Meeting Eligibility Criteria

eTable 3. Baseline Demographic and Socioeconomic Characteristics of Participants in the 28 Included Studies

eTable 4. Excluded Studies From the Original Search With Reasons for Exclusion

eTable 5. Excluded Studies That Were Eliminated From the Citation Chasing With Corresponding Rationale

eTable 6. Subgroup Analysis Showing Change in SBP at 6 Months

eTable 7. Subgroup Analysis Showing Change in DBP at 6 Months

eTable 8. Subgroup Analysis Showing SBP Values at 6 Months

eTable 9. Subgroup Analysis Showing DBP Values at 6 Months

eFigure 1. Differences in Diastolic Blood Pressure Changes From Baseline to Follow-Up Time Points Between Digital Health Intervention and Control Groups

eFigure 2. Differences in Follow-Up Diastolic Blood Pressures Between Digital Health Intervention and Control Groups at Different Time Points

eFigure 3. Sensitivity Analysis for Presence of Publication Bias Using Change in SBP Outcome at 6-Month Follow-Up and Leave-One-Out Meta-Analysis Results for Same Outcome

eFigure 4. Sensitivity Analysis for Presence of Publication Bias Using Follow-Up SBP Value Outcome at 6-Month Follow-up and Leave-One-Out Meta-Analysis Results for Same Outcome

eFigure 5. Sensitivity Analysis for Presence of Publication Bias Using Change in DBP Outcome at 6-Month Follow-Up and Leave-One-Out Meta-Analysis Results for Same Outcome

eFigure 6. Sensitivity Analysis for Presence of Publication Bias Using Follow-Up DBP Value Outcome at 6-Month Follow-Up and Leave-One-Out Meta-Analysis Results for Same Outcome

eFigure 7. Risk of Bias Assessment for Included Studies

This supplementary material has been provided by the authors to give readers additional information about their work.

© 2024 Katz ME et al. *JAMA Network Open*.

eTable 1. Search strategies and additional methodology

**Search for Ovid Embase**<br>1 exp mobile application/<br>2 exp wearable computer/<br>3 ((mobile or cell phone or cellular or smartphone or iphone or android or windows or blackberry or handheld or portable or ipad or tablet or phone or telephone) adj3 (app or apps or application* or health or device* or message* or intervention*)).mp.<br>4 (health adj3 (app or apps or application* or device*)).mp.<br>5 (mHealth or telemedicine or wearable or mobile health or digital treatment* or digital health or ehealth or e-health or m-health or smartwatch* or smart watch* or tele monitor* or telemonitor* or mobile technolog* or text message* or SMS).mp.<br>6 1 or 2 or 3 or 4 or 5<br>7 exp hypertension/<br>8 ((high or elevated or raised) adj3 blood pressure*).mp.<br>9 (hypertension or hypertensive).mp.<br>10 (blood pressure adj3 (manage* or control* or prevent* or monitor*)).mp.<br>11 7 or 8 or 9 or 10<br>12 6 and 11<br>13 exp socioeconomics/<br>14 "social determinants of health"/<br>15 health disparity/<br>16 health care disparity/<br>17 minority health/<br>18 health equity/<br>19 (low* income or low*-income or impoverish* or poverty or homeless or disadvantaged or disenfranchis* or financial* distress* or financial* stress* or economic factor* or socioeconomic* or welfare* or subsidized or economic burden* or underprivilege* or destitute or bankrupt* or poverty-stricken).mp.<br>20 (social determinant* or implicit attitude* or implicit bias* or health bias* or racial bias* or conscious attitude* or unconscious bias* or nonconscious bias* or non-conscious bias* or conscious bias*).mp.<br>21 (prejudice* or inequal* or inequit* or disparit* or equalit* or equit* or unequal* or discrimination or injustice* or justice* or racist* or racism* or stigma* or sterotyp*).mp.<br>22 ((minitor* or structural* or cultural* or multi-cultur* or multicultur* or linguistic* or social* or intercultural* or inter-cultural* or cross-cultural or crosscultural or multi-rac* or multi-ethnic* or multiethnic* or multi-lingual or multilingual or trans-cultural or transcultural or ethnocultur* or ethno-cultur* or socio-cultural or sociocultural or bi-rac* or birac*) adj3 (status* or competen* or awareness* or diversit* or discrimin* or bias*)).mp.<br>23 or/13-22<br>24 12 and 23
**Search for Ovid Medline**<br>1 Mobile Applications/<br>2 exp Wearable Electronic Devices/<br>3 ((mobile or cell phone or cellular or smartphone or iphone or android or windows or blackberry or handheld or portable or ipad or tablet or phone or telephone) adj3 (app or apps or application* or health or device* or message* or intervention*)).mp.<br>4 (health adj3 (app or apps or application* or device*)).mp.<br>5 (mHealth or telemedicine or wearable or mobile health or digital treatment* or digital health or ehealth or e-health or m-health or smartwatch* or smart watch* or tele monitor* or telemonitor* or mobile technolog* or text message* or SMS).mp.<br>6 1 or 2 or 3 or 4 or 5<br>7 exp Hypertension/<br>8 ((high or elevated or raised) adj3 blood pressure*).mp.<br>9 (hypertension or hypertensive).mp.<br>10 (blood pressure adj3 (manage* or control* or prevent* or monitor*)).mp.<br>11 7 or 8 or 9 or 10<br>12 6 and 11

13	exp Socioeconomic Factors/
14	"Social Determinants of Health"/
15	health status disparities/
16	Healthcare Disparities/
17	Minority Health/
18	Health Equity/
19	(low* income or low*-income or impoverish* or poverty or homeless or disadvantaged or disenfranchis* or financial* distress* or financial* stress* or economic factor* or socioeconomic* or welfare* or subsidized or economic burden* or underprivilege* or destitute or bankrupt* or poverty-stricken).mp.
20	(social determinant* or implicit attitude* or implicit bias* or health bias* or racial bias* or conscious attitude* or unconscious bias* or nonconscious bias* or non-conscious bias* or conscious bias*).mp.
21	((minorit* or structural* or cultural* or multi-cultur* or multicultur* or linguistic* or social* or intercultural* or inter-cultural* or cross-cultural or crosscultural or multi-rac* or multi-ethnic* or multiethnic* or multi-lingual or multilingual or trans-cultural or transcultural or ethnocultur* or ethno-cultur* or socio-cultural or sociocultural or bi-rac* or birac*) adj3 (status* or competen* or awareness* or diversit* or discrimin* or bias*)).mp.
22	13 or 14 or 15 or 16 or 17 or 18 or 19 or 20 or 21
23	12 and 22

Search for Web of Science Core Collection

#1 TS=("low* income" or "low*-income" or impoverish* or poverty or homeless or disadvantaged or disenfranchis* or "financial* distress*" or "financial* stress*" or "economic factor*" or socioeconomic* or welfare* or subsidized or "economic burden*" or underprivilege* or destitute or bankrupt* or "poverty-stricken") or TS=("social determinant*" or "implicit attitude*" or "implicit bias*" or "health bias*" or "racial bias*" or "conscious attitude*" or "unconscious bias*" or "nonconscious bias*" or "non-conscious bias*" or "conscious bias*") or TS=((minorit* or structural* or cultural* or multi-cultur* or multicultur* or linguistic* or social* or intercultural* or inter-cultural* or cross-cultural or crosscultural or multi-rac* or multi-ethnic* or multiethnic* or multi-lingual or multilingual or trans-cultural or transcultural or ethnocultur* or ethno-cultur* or socio-cultural or sociocultural or bi-rac* or birac*) near/3 (status* or competen* or awareness* or diversit* or discrimin* or bias*))

#2 TS=((high or elevated or raised) near/3 "blood pressure*") or TS=(hypertension or hypertensive) or TS=("blood pressure" near/3 (manage* or control* or prevent* or monitor*))

#3 TS=((mobile or "cell phone" or cellular or smartphone or iphone or android or windows or blackberry or handheld or portable or ipad or tablet or phone or telephone) near/3 (app or apps or application* or health or device* or message* or intervention*)) or TS=(health near/3 (app or apps or application* or device*)) or TS=(mHealth or telemedicine or wearable or "mobile health" or "digital treatment*" or "digital health" or ehealth or e-health or m-health or smartwatch* or "smart watch*" or "tele monitor*" or telemonitor* or "mobile technolog*" or "text message*" or SMS)

#4 #1 and #2 and #3

Search for Scopus

(TITLE-ABS-KEY ("low* income" OR "low*-income" OR impoverish* OR poverty OR homeless OR disadvantaged OR disenfranchis* OR "financial* distress*" OR "financial* stress*" OR "economic factor*" OR socioeconomic* OR welfare* OR subsidized OR "economic burden*" OR underprivilege* OR destitute OR bankrupt* OR "poverty-stricken") OR TITLE-ABS-KEY ("social determinant*" OR "implicit attitude*" OR "implicit bias*" OR "health bias*" OR "racial bias*" OR "conscious attitude*" OR "unconscious bias*" OR "nonconscious bias*" OR "non-conscious bias*" OR "conscious bias*") OR TITLE-ABS-KEY ((minorit* OR structural* OR cultural* OR multi-cultur* OR multicultur* OR linguistic* OR social* OR intercultural* OR inter-cultural* OR cross-cultural OR crosscultural OR multi-rac* OR multi-ethnic* OR multiethnic* OR multi-lingual OR multilingual OR trans-cultural OR transcultural OR ethnocultur* OR ethno-cultur* OR socio-cultural OR sociocultural OR bi-rac* OR birac*) W/3 (status* OR competen* OR awareness* OR diversit* OR discrimin* OR bias*))) AND (TITLE-ABS-KEY ((high OR elevated OR raised) W/3 "blood pressure*") OR TITLE-ABS-KEY (hypertension OR hypertensive) OR TITLE-ABS-KEY ("blood pressure" W/3 (manage* OR control* OR prevent* OR monitor*))) AND (TITLE-ABS-KEY ((mobile OR "cell

© 2024 Katz ME et al. *JAMA Network Open*.

phone" OR cellular OR smartphone OR iphone OR android OR windows OR blackberry OR handheld OR portable OR ipad OR tablet OR phone OR telephone) W/3 (app OR apps OR application* OR health OR device* OR message* OR intervention*)) OR TITLE-ABS-KEY (health W/3 (app OR apps OR application* OR device*)) OR TITLE-ABS-KEY (mhealth OR telemedicine OR wearable OR "mobile health" OR "digital treatment*" OR "digital health" OR ehealth OR e-health OR m-health OR smartwatch* OR "smart watch*" OR "tele monitor*" OR telemonitor* OR "mobile technolog*" OR "text message*" OR sms))
**Search for Google Scholar**<br>hypertension mhealth
**Search for Pubmed**<br>((low* income or low*-income or impoverish* or poverty or homeless or disadvantaged or disenfranchis* or financial* distress* or financial* stress* or economic factor* or socioeconomic* or welfare* or subsidized or economic burden* or underprivilege* or destitute or bankrupt* or poverty-stricken or social determinant* or implicit attitude* or implicit bias* or health bias* or racial bias* or conscious attitude* or unconscious bias* or nonconscious bias* or non-conscious bias* or conscious bias*) AND (high blood pressure* or elevated blood pressure* or raised blood pressure* or hypertension or hypertensive or blood pressure monitor* or blood pressure control* or blood pressure prevent*)) AND ((mHealth or telemedicine or wearable or mobile health or digital treatment* or digital health or ehealth or e-health or m-health or smartwatch* or smart watch* or tele monitor* or telemonitor* or mobile technolog* or text message* or SMS or health app*))
**Search for Cochrane Library**<br>#1 ("low* income" or "low*-income" or impoverish* or poverty or homeless or disadvantaged or disenfranchis* or "financial* distress*" or "financial* stress*" or "economic factor*" or socioeconomic* or welfare* or subsidized or "economic burden*" or underprivilege* or destitute or bankrupt* or "poverty-stricken"):ti,ab or ("social determinant*" or "implicit attitude*" or "implicit bias*" or "health bias*" or "racial bias*" or "conscious attitude*" or "unconscious bias*" or "nonconscious bias*" or "non-conscious bias*" or "conscious bias*"):ti,ab or ((minorit* or structural* or cultural* or multi-cultur* or multicultur* or linguistic* or social* or intercultural* or inter-cultural* or cross-cultural or crosscultural or multi-rac* or multi-ethnic* or multiethnic* or multi-lingual or multilingual or trans-cultural or transcultural or ethnocultur* or ethno-cultur* or socio-cultural or sociocultural or bi-rac* or birac*) near/3 (status* or competen* or awareness* or diversit* or discrimin* or bias*)):ti,ab<br>#2 ((high or elevated or raised) near/3 "blood pressure*"):ti,ab or (hypertension or hypertensive):ti,ab or ("blood pressure" near/3 (manage* or control* or prevent* or monitor*)):ti,ab<br>#3 ((mobile or "cell phone" or cellular or smartphone or iphone or android or windows or blackberry or handheld or portable or ipad or tablet or phone or telephone) near/3 (app or apps or application* or health or device* or message* or intervention*)):ti,ab or (health near/3 (app or apps or application* or device*)):ti,ab or (mHealth or telemedicine or wearable or "mobile health" or "digital treatment*" or "digital health" or ehealth or e-health or m-health or smartwatch* or "smart watch*" or "tele monitor*" or telemonitor* or "mobile technolog*" or "text message*" or SMS):ti,ab<br>#4 #1 and #2 and #3

© 2024 Katz ME et al. *JAMA Network Open*.

eTable 2. Additional study characteristics for the included studies (n=28) meeting our eligibility criteria

Study	BP as Primary Outcome	Study Setting / Community Engagement	Study Duration	Trial Name, if applicable
Naqvi, 2022	No, feasibility	Comprehensive Stroke Center that serves a majority of Hispanic low-income community.	2020-2021	Telehealth After Stroke Care (TASC) trial
Brewer, 2022	No, LS7 score	Community-based participatory research; Community Steering Committee provided study oversight to ensure its community centeredness; community input through an iterative, semi structured focus group series with African American community members affiliated with partnering African American churches	2019-2020	FAITH! Trial (Fostering African American Improvement in Total Health)
Clark, 2021	Yes	N/A	2018-2020	N/A
Schoenthaler, 2020	No, feasibility	Safety net primary care clinic in New York City, which serves a predominately diverse, low-income urban patient population	2016-2018	N/A
Vaughan, 2020	Yes	Nonprofit clinic serving primarily low-income and uninsured individuals	N/A	A Telehealth-supported, Integrated care with CHWs, and MEdication-access (TIME)
Zha, 2020	Yes	Local community health center	2016-2017	Improve Hypertension Monitoring and Self-management by Using mHealth
Schroeder, 2020	Yes	Urban Indian Health Organization; First Nations Community HealthSource (FNCH), a UIHO that provides community-based health and human services to AI/AN and other vulnerable populations	2017-2019	Controlling Hypertension in Native American and Other Populations
Still, 2020	Yes	Affiliated community clinics, as well as from community outreach and engagements; CCBN nurses serve as a bridge between underserved communities and their healthcare needs	2018-2019	Technology-based Intervention and Positive Psychological Training for Blood Pressure Control in African Americans (TechSupport)
Persell, 2020	Yes	Outpatient clinics affiliated with a large health care system	2017-2019	The Smart Hypertension Control Study (iSmartHyp)
Chandler, 2019	Yes	N/A	N/A	Smartphone Med Adherence Stops Hypertension, SMASH
Bennett, 2018	No, weight change	Community health center system serving primarily racial/ethnic minority patients and those with a low-income and/or lack of insurance	2013-2015	New Media Obesity Treatment in Community Health Centers
Bosworth, 2018	No, CVD risk score	N/A -- VA Health System	2011-2015	The CITIES Trial

© 2024 Katz ME et al. *JAMA Network Open*.

Skolarus, 2018	Yes	Reach Out was a collaborative program given it was both faith placed, with the church involved in recruiting and delivering the intervention and faith based initiative, with church health team involvement. Community and academic partners, church health teams and church leadership, including pastors and elders, were present for the recruitment events.	2014-2015	Community Study to Reduce High Blood Pressure Through Text Messaging (REACH OUT)
Morawski, 2018	Yes	N/A	2016-2017	Medication Adherence Improvement Support App For Engagement - Blood Pressure (MedISAFE-BP)
Fortmann, 2017	No, HbA1C	Participant recruitment from clinic sites within a network of federally qualified health centers serving racial/ethnic minority and low-income individuals	2012-2014	Dulce Digital Study
Frias, 2017	Yes	Multiple outpatient primary care sites	2015-2016	Proteus Discover in Subjects With Uncontrolled Hypertension and Type 2 Diabetes
Tuot, 2016	Yes	2 primary care clinics (1 academic training site and 1 community clinic) in a public health care delivery system	2013-2015	Kidney Awareness Registry and Education (KARE)
Bove, 2013	Yes	Participant recruitment through advertisement and communication at local churches and community centers	2008-2010	Using a Telemedicine System to Promote Patient Care Among Underserved Individuals (AHRQ)
Crowley, 2013	Yes	Multiple clinics where PCPs care for a large proportion of African-American patients; cultural sensitivity training for research staff	2008-2011	The CHANGE Study
Rifkin, 2013	No, mHealth acceptability	N/A -- VA Health System	N/A	N/A
Margolis, 2013	Yes	Multispecialty practice part of an integrated health system	2009-2012	Home Blood Pressure Telemonitoring and Case Management to Control Hypertension (Hyperlink)
Bennett, 2012	No, weight change	Multiple urban community health centers that serving primarily racial/ethnic minority patients; provided tailored information on community resources for healthy behaviors; trained community health educators	2008-2011	Be Fit, Be Well
McKee, 2011	Yes	Federally qualified health center; trained home health nurses	N/A	N/A
Frosch, 2011	No, HbA1C	Multiple academic primary care practices and one community-based safety net clinics caring for poor and uninsured patients	2008-2010	Improving Diabetes Care With Patient Decision Aids - A Trial in Community-based Primary Care
Anderson	No	Community health center serving predominantly Hispanic individuals and those with a low-income and lack of insurance	N/A	Managing the Space Between Visits (MSBV)
Brennan, 2010	Yes	DM nurses received cultural competency training	2006-2007	N/A

© 2024 Katz ME et al. *JAMA Network Open*.

Bosworth, 2009	Yes	Multiple university-affiliated primary care clinics	2004-2008	Take Control of Your Blood Pressure (TCYB)
Shea, 2009	Yes	Primary care practices	2000-2007	Randomized Trial of Telemedicine for Diabetes Care (IDEATel)

© 2024 Katz ME et al. *JAMA Network Open*.

eTable 3. Baseline demographic and socioeconomic characteristics of participants in the included studies (n=28)

Author, year	Sample Size	Mean (SD) Age, years	Women n (%)	Black n (%)	Hispanic n (%)	Education <HS n (%)	Low Income n (%)	Medicaid n (%)	Medicare n (%)	Uninsured n (%)
Naqvi, 2022[54]	50	64.3 (14.0)	18 (36.0)	16 (32.0)	22 (44.0)	16 (32.0)	–	14 (28.0)	13 (26.0)	4 (8.0)
Brewer, 2022[50]	68	54.2 (12.3)	48 (70.6)	68 (100.0)	–	7 (10.6)	11 (19.0)	–	–	8 (12.1)
Clark, 2021[29]	991	59.9 (14.2)	662 (66.5)	549 (56.5)	–	33 (27.5)	56 (46.7)	–	–	–
Schoenthaler, 2020[55]	42	57.6 (11.1)	19 (45.2)	42 (100.0)	–	5 (11.9)	26 (62.8)	19 (45.3)	7 (16.7)	8 (19.0)
Vaughan, 2020[45]	89	54.9 (8.1)	64 (71.9)	–	89 (100.0)	–	89 (100.0)	–	–	–
Zha, 2020[56]	25	52.2 (6.6)	22 (88.0)	24 (96.0)	1 (4.0)	–	–	–	–	–
Schroeder, 2020[32]	295	53.4 (11.3)	176 (59.7)	14 (4.8)	153 (51.9)	111 (37.8)	213 (72.2)	46 (15.6)	46 (15.6)	2 (0.7)
Still, 2020[42]	60	59.5 (8.9)	45 (75.0)	60 (100.0)	–	–	16 (26.7)	–	–	–
Persell, 2020[57]	297	59.0 (12.8)	182 (61.3)	103 (34.7)	23 (7.7)	5 (1.7)	–	–	–	–
Tuot, 2019[58]	137	58.0 (10.4)	71 (51.8)	58 (42.3)	50 (36.5)	46 (33.6)	–	61 (44.5)	37 (27.0)	39 (28.4)
Chandler, 2019[59]	54	45.6 (7.7)	36 (66.7)	–	54 (100.0)	39 (72.0)	36 (66.5)	–	–	–
Bennett, 2018[60]	351	50.7 (8.9)	239 (68.0)	183 (52.0)	44 (13.0)	51 (15.0)	180 (51.0)	–	–	175 (50.0)
Bosworth, 2018[61]	428	61.2 (8.7)	65 (15.2)	213 (50.0)	14 (3.3)	128 (29.9)	–	–	–	–
Skolarus, 2018[43]	94	58 (9.8)	74 (79.0)	91 (97.0)	–	–	–	21 (22.0)	15 (16.0)	7 (7.0)
Morawski, 2018[62]	411	52.1 (10.3)	247 (60.1)	103 (25.1)	–	8 (1.9)	–	–	–	–
Fortmann, 2017[30]	126	48.5 (9.8)	94 (74.5)	–	126 (100.0)	90 (73.0)	109 (87.5)	–	–	95 (75.5)
Frias, 2017[63]	109	59.4 (13.6)	55 (50.5)	17 (15.6)	51 (46.8)	34 (31.2)	62 (56.9)	–	–	–
Bove, 2013[47]	241	59.6 (13.6)	157 (65.1)	195 (80.9)	7 (2.9)	39 (16.2)	128 (53.1)	37 (15.4)	82 (34.0)	13 (5.4)
Crowley, 2013[44]	359	56.5 (12.0)	258 (72.0)	359 (100.0)	–	108 (30.0)	135 (37.6)	59 (16.5)	147 (40.9)	21 (5.8)
Rifkin, 2013[64]	43	68.3 (7.9)	2 (4.7)	11 (25.6)	–	–	–	–	–	–
Margolis, 2013[31]	450	61.1 (12.0)	201 (44.7)	53 (11.8)	10 (2.2)	76 (17.4)	65 (17.0)	–	–	–
Bennett, 2012[46]	365	54.6 (10.9)	250 (68.5)	260 (71.2)	48 (13.2)	120 (32.9)	199 (54.5)	123 (33.7)	75 (20.5)	–
McKee, 2011[65]	55	60.0 (9.7)	36 (66.7)	13 (23.6)	40 (72.7)	26 (47.3)	40 (72.7)	34 (61.8)	–	4 (7.3)
Frosch, 2011[48]	201	55.5 (8.6)	97 (48.5)	32 (16.1)	112 (55.7)	115 (57.0)	174 (86.5)	–	–	136 (67.7)
Anderson, 2010[49]	295	–	171 (58.0)	27 (9.2)	223 (75.6)	260 (88.1)	–	126 (42.7)	72 (24.4)	44 (14.9)
Brennan, 2010[66]	638	55.7 (11.5)	427 (66.9)	638 (100.0)	–	39 (5.2)	256 (40.1)	–	–	–
Bosworth, 2009[67]	318	61.5 (12.0)	200 (62.9)	154 (48.5)	–	113 (35.5)	60 (18.9)	–	–	–
Shea, 2009[68]	1,665	70.8 (6.6)	1,046 (62.8)	248 (14.9)	586 (35.2)	–	–	651 (39.1)	1,665 (100.0)	–
**Pooled**	**8,257**	**57.2 (10.4)**	**4,962 (60.1)**	**3,531 (42.8)**	**1,631 (19.8)**	**1,471 (17.8)**	**1,884 (22.8)**	**1,177 (14.3)**	**2,146 (26.0)**	**548 (6.6)**

© 2024 Katz ME et al. *JAMA Network Open*.

eTable 4. Excluded studies from the original search with reasons for exclusion.

First Author's Last Name	Year	Title	Journal	Reason for Exclusion
Abughosh	2015	Impact of a pharmacist telephone intervention on medication adherence among hypertensive patients with diabetes in a medicare advantage plan	Value in Health	Wrong outcomes
Abughosh	2016	A Pharmacist Telephone Intervention to Identify Adherence Barriers and Improve Adherence Among Nonadherent Patients with Comorbid Hypertension and Diabetes in a Medicare Advantage Plan	Journal of Managed Care & Specialty Pharmacy	Wrong outcomes
Actrn	2020	Home-based blood pressure monitoring of Hypertensive Diseases in Pregnancy - A randomised controlled trial (REMOTE CONTROL trial)	Clinicaltrials.gov	Protocol
Afarideh	2016	Text message support for weight loss in patients with prediabetes: A randomized clinical trial. Diabetes care 2016;39:1364-1370	Diabetes Care	No original data
Agarwal	2019	Closing Gaps in Cardiovascular Disease Prevention Through Community-Centered Primary Health Care Interventions	JAMA Cardiology	No original data
Agnihothri	2021	Mobile health application usage and quality of care at a hypertension clinic: an observational cohort study	Journal of Hypertension	Insufficient data
Aikens	2014	Diabetes self-management support using mHealth and enhanced informal caregiving	Journal of Diabetes and its Complications	Wrong outcomes
Albini	2016	An ICT and mobile health integrated approach to optimize patients' education on hypertension and its management by physicians: the Patients Optimal Strategy of Treatment(POST) pilot study	Conference proceedings :	Ineligible patient population
Allen	2019	SMS-facilitated home blood pressure monitoring: A qualitative analysis of resultant health behavior change	Patient Education and Counseling	Wrong outcomes
Anthony	2015	Outpatient blood pressure monitoring using bi-directional text messaging	Journal of the American Society of Hypertension	Wrong outcomes
Arkerson	2023	Remote Monitoring Compared with In-Office Surveillance of Blood Pressure in Patients with Pregnancy-Related Hypertension: A Randomized Controlled Trial	Obstetrics and Gynecology	Wrong patient population
Arora	2012	A mobile health intervention for inner city patients with poorly controlled diabetes: proof-of-concept of the TExT-MED program	Diabetes technology & therapeutics	Wrong outcomes
Asgary	2015	Perceptions, attitudes, and experience regarding mHealth among homeless persons in New York City shelters	Journal of health communication	Wrong study design
Ayala	2017	Environmental scan of telemedicine networks with services for hypertension management, 2016	Circulation: Cardiovascular Quality and Outcomes.	Wrong outcomes
Baggett	2018	Cardiovascular disease and homelessness	Journal of the American College of Cardiology	No original data
Baig	2010	A randomized community-based intervention trial comparing faith community nurse referrals to telephone-assisted physician appointments for health fair participants with elevated blood pressure	J Gen Intern Med	Wrong intervention

© 2024 Katz ME et al. *JAMA Network Open*.

Banala	2023	Improving Hypertension Management through a Deviceless Remote Monitoring Program at a Safety-Net Resident Continuity Clinic	Journal of General Internal Medicine	Insufficient data
Becker	2014	mHealth 2.0: experiences, possibilities, and perspectives	JMIR mHealth and uHealth	No original data
Becker	2016	Feasibility of mhealth technology use among a sample of isolated rural men at high risk for cardiovascular disease	Cardiopulmonary Physical Therapy Journal	Wrong outcomes
Bennett	2009	The effectiveness of health coaching, home blood pressure monitoring, and home-titration in controlling hypertension among low-income patients: protocol for a randomized controlled trial	BMC Public Health	Protocol
Beran	2018	Key components of success in a randomized trial of blood pressure telemonitoring with medication therapy management pharmacists	J Am Pharm Assoc	No original data
Black	2014	A remote monitoring and telephone nurse coaching intervention to reduce readmissions among patients with heart failure: study protocol for the Better Effectiveness After Transition - Heart Failure (BEAT-HF) randomized controlled trial	Trials	Protocol
Blixen	2018	Patient participation in the development of a customized m-Health intervention to improve medication adherence in poorly adherent individuals with bipolar disorder (BD) and hypertension (HTN)	International journal of healthcare	Wrong study design
Blixen	2018	Patient participation in the development of a customized m-Health intervention to improve medication adherence in poorly adherent individuals with bipolar disorder (BD) and hypertension (HTN)	International journal of healthcare	Duplicate
Blood	2023	Results of a Remotely Delivered Hypertension and Lipid Program in More Than 10000 Patients Across a Diverse Health Care Network	Jama Cardiology	Wrong patient population
Boden-Albala	2019	Efficacy of a Discharge Educational Strategy vs Standard Discharge Care on Reduction of Vascular Risk in Patients With Stroke and Transient Ischemic Attack The DESERVE Randomized Clinical Trial	Jama Neurology	Wrong intervention
Bosworth	2011	Racial differences in two self-management hypertension interventions	American Journal of Medicine	No original data
Bowsworth	2011	Home blood pressure management and improved blood pressure control: results from a randomized controlled trial	Arch Intern Med	No original data
Bove	2011	Reducing cardiovascular disease risk in medically underserved urban and rural communities	American Heart Journal	No original data
Bove	2013	Managing hypertension in urban underserved subjects using telemedicine: A clinical trial	Journal of the American College of Cardiology	Insufficient data
Breil	2019	Acceptance of mHealth apps for self-management among people with hypertension	GMDS	Ineligible patient population
Brewer	2023	mHealth Intervention for Promoting Hypertension Self-management Among African American Patients Receiving Care at a Community Health Center: Formative Evaluation of the FAITH! Hypertension App	JMIR Form Res	No original data
Brewer	2018	Community-based, cluster-randomized pilot trial of a cardiovascular mHealth intervention: Rationale, design, and baseline findings of the FAITH! Trial	American Heart Journal	Insufficient data

© 2024 Katz ME et al. *JAMA Network Open*.

Brewer	2018	mHealth intervention promoting cardiovascular health among African-Americans: recruitment and baseline characteristics of a pilot study	JMIR Research Protocols	Protocol
Brook	2022	Utilizing Mobile Health Units for Mass Hypertension Screening in Socially Vulnerable Communities Across Detroit	Hypertension	Wrong study design
Brown	2019	"Drugs Do Not Work on Patients Who Do Not Take Them" Can We Do Better in Patient Adherence?	Journal of Cardiac Failure	No original data
Buckley	2015	Corrections clinic	Annals of Internal Medicine	No original data
Buis	2020	Understanding the Feasibility, Acceptability, and Efficacy of a Clinical Pharmacist-led Mobile Approach (BPTrack) to Hypertension Management: Mixed Methods Pilot Study	Journal of Med Internet Res	
Buis	2017	Text Messaging to Improve Hypertension Medication Adherence in African Americans From Primary Care and Emergency Department Settings: Results From Two Randomized Feasibility Studies	JMIR mHealth and uHealth	Wrong comparator
Buis	2019	Improving Blood Pressure Among African Americans With Hypertension Using a Mobile Health Approach (the MI-BP App): Protocol for a Randomized Controlled Trial	JMIR Research Protocols	Protocol
Blyler	2021	Improving efficiency of the barbershop model of hypertension care for black men with virtual visits	Journal of the American Heart Association	Wrong comparator
Cardozo	2010	Telemedicine for recently discharged older patients	Telemedicine Journal & E-Health	Wrong outcomes
Castelnuovo	2015	Chronic care management of globesity: promoting healthier lifestyles in traditional and mHealth based settings	Frontiers in psychology	No original data
Catov	2016	Pregnancy as a window to future health: short-term costs and consequences	American Journal of Obstetrics and Gynecology	No original data
Chad-Friedman	2018	Total Lifestyle Coaching: A Pilot Study Evaluating the Effectiveness of a Mind-Body and Nutrition Telephone Coaching Program for Obese Adults at a Community Health Center	Glob Adv Health Med	Wrong intervention
Choi	2014	Effectiveness of telemedicine: videoconferencing for low-income elderly with hypertension	Telemedicine Journal & E-Health	Ineligible patient population
Chowdhury	2018	Effectiveness of telehealth on hypertension management and control among disparate populations-a systematic review and meta-analysis	Circulation: Cardiovascular Quality and Outcomes. Conference: American Heart Association Quality of Care and Outcomes Research Scientific Sessions, QCOR	No original data
Clark	2020	REMOTE HYPERTENSION MANAGEMENT USING BLOOD PRESSURE TELEMONITORING IN A RURAL AND LOW-INCOME POPULATION	Journal of the American College of Cardiology	Insufficient data
Clark	2020	Digital Informed Consent in a Rural and Low-Income Population	JAMA Cardiology	Wrong outcomes

© 2024 Katz ME et al. *JAMA Network Open*.

Cole	2017	Community-Based, Preclinical Patient Navigation for Colorectal Cancer Screening Among Older Black Men Recruited From Barbershops: The MISTER B Trial	American journal of public health	Wrong outcomes
Crowley	2016	Practical Telemedicine for Veterans with Persistently Poor Diabetes Control: A Randomized Pilot Trial	Telemed J E Health	No original data
Daraei	2015	Mobile health screening initiatives: a narrative of three unique programs in underserved populations	Innovation and Entrepreneurship in Health	No original data
Dary	2014	Remote monitoring in heart failure: Feasibility and results of a limited 14-day follow-up of 83 patients	European Research in Telemedicine	Ineligible patient population
David	2023	Effect of Mobile Health Interventions on Lifestyle and Anthropometric Characteristics of Uncontrolled Hypertensive Participants: Secondary Analyses of a Randomized Controlled Trial	Healthcare Basel	Wrong patient population
Davidson	2015	Evaluation of an mHealth Medication Regimen Self-Management Program for African American and Hispanic Uncontrolled Hypertensives	J Pers Med	Insufficient data
DeNicola	2020	Connected health-enabled remote blooding pressure monitoring of low-risk pregnancy in rural setting	Obstetrics and Gynecology	Wrong outcomes
Desai	2016	Smartphone apps: A patient's new best friend?	Clinical Journal of the American Society of Nephrology	No original data
Dicianno	2015	Perspectives on the evolution of mobile (mHealth) technologies and application to rehabilitation	Physical therapy	No original data
Donahue	2016	Lessons Learned From Implementing Health Coaching in The Heart Healthy Lenoir Hypertension Study	Progress in Community Health Partnerships-Research Education and Action	Wrong outcomes
Dorsch	2020	Effects of a Novel Contextual Just-In-Time Mobile App Intervention (LowSalt4Life) on Sodium Intake in Adults With Hypertension: Pilot Randomized Controlled Trial	JMIR mHealth and uHealth	Wrong intervention
Duan	2020	Using goal-directed design to create a mobile health app to improve patient compliance with hypertension self-management: development and deployment	JMIR mHealth and uHealth	Ineligible patient population
Dunsmuir	2014	Development of mHealth applications for pre-eclampsia triage	IEEE journal of biomedical and health informatics	No original data
Eccher	2014	A Mobile Logbook to Diagnose Masked Hypertension: a Pilot Application	E-Health - for Continuity of Care	Wrong outcomes
Egede	2017	Randomized Controlled Trial of Technology-Assisted Case Management in Low Income Adults with Type 2 Diabetes	Diabetes Technology and Therapeutics	Wrong outcomes
ElHadidi	2020	Evidence beyond the digital medication pill	European Heart Journal - Cardiovascular Pharmacotherapy	No original data
Ferdinand	2023	TEXT MY BP MEDS NOLA: A pilot study of text-messaging and social support to increase hypertension medication adherence	American Heart Journal	Insufficient data

© 2024 Katz ME et al. *JAMA Network Open*.

Florin	1971	Heart screening in the Newark Model Cities area: a feasibility study	American journal of public health	Wrong intervention
Foley	2012	Weight gain prevention among black women in the rural community health center setting: the Shape Program	BMC Public Health	Protocol
Foley	2016	Track: A randomized controlled trial of a digital health obesity treatment intervention for medically vulnerable primary care patients	Contemporary Clinical Trials	Protocol
Fundoiano-Hershcovitz	2022	Blood Pressure Monitoring as a Digital Health Tool for Improving Diabetes Clinical Outcomes: Retrospective Real-world Study	Journal of Medical Internet Research	Insufficient data
Ganti	2021	Enabling Wearable Pulse Transit Time-Based Blood Pressure Estimation for Medically Underserved Areas and Health Equity: Comprehensive Evaluation Study	JMIR mHealth and uHealth	Wrong study design
Gasper	2020	Instituting video visits for hypertension control in an urban teaching practice	Journal of General Internal Medicine	Insufficient data
Gerin	2007	The medication Adherence and Blood Pressure Control (ABC) trial: A multi-site randomized controlled trial in a hypertensive, multi-cultural, economically disadvantaged population	Contemporary Clinical Trials	Protocol
Girma	2021	Self-measured blood pressure monitoring (SBPM) during the COVID-19 pandemic	Journal of General Internal Medicine	Insufficient data
Gollamudi	2016	A framework for smartphone-enabled, patient-generated health data analysis	PeerJ	Insufficient data
Gong	2020	Mobile health applications for the management of primary hypertension: A multicenter, randomized, controlled trial	Medicine	Ineligible patient population
Gonzalez	2020	Design and methods of NYC care calls: An effectiveness trial of telephone-delivered type 2 diabetes self-management support	Contemp Clin Trials	Protocol
Gopalan	2015	Health-specific information and communication technology use and its relationship to chronic disease status in communities on the South side of Chicago	Journal of General Internal Medicine	Wrong outcomes
Greer	2022	Exploring Feasibility of mHealth to Manage Hypertension in Rural Black Older Adults: A Convergent Parallel Mixed Method Study	Patient Prefer Adherence	Insufficient data
Grobbee	2017	Secondary prevention of cardiovascular disease: Unmet medical need, implementation and innovation	European Journal of Preventive Cardiology	No original data
Gulliford	2019	Digital health intervention at older ages	The Lancet Digital Health	No original data
Gyselaers	2019	Mobile Health Applications for Prenatal Assessment and Monitoring	Curr Pharm Des	No original data
Hampton	2012	Recent advances in mobile technology benefit global health, research, and care	JAMA - Journal of the American Medical Association	No original data
Harrison	2013	A randomized controlled trial of an automated telephone intervention to improve blood pressure control	Journal of Clinical Hypertension	Wrong intervention
Hauspurg	2020	25: The impact of race on blood pressure trajectory in the postpartum period	American Journal of Obstetrics and Gynecology	Insufficient data

© 2024 Katz ME et al. *JAMA Network Open*.

Heisler	2019	Effectiveness of Technologically Enhanced Peer Support in Improving Glycemic Management Among Predominantly African American, Low-Income Adults With Diabetes	Diabetes Educ	Insufficient data
Hernandez	2020	A Web-Based Positive Psychological Intervention to Improve Blood Pressure Control in Spanish-Speaking Hispanic/Latino Adults With Uncontrolled Hypertension: Protocol and Design for the Alegrate! Randomized Controlled Trial	JMIR Research Protocols	Protocol
Herzallah	2019	Combined patient-provider engagement system and mhealth interventions improved medication adherence for minority and low-income patients with diabetes compared to mhealth only: The office-guidelines applied to practice program	Journal of General Internal Medicine	Wrong outcomes
Hines	2022	Community Hypertension Screening and Care Referral With Blood Pressure-Measuring Kiosks, Digital Education Modalities, and Text Messages	American journal of hypertension	No original data
Hirshberg	2019	Text message remote monitoring reduced racial disparities in postpartum blood pressure ascertainment	American Journal of Obstetrics & Gynecology	Wrong outcomes
Hirshberg	2019	Text message remote blood pressure monitoring eliminated racial disparities in postpartum hypertension care	American Journal of Obstetrics and Gynecology	Wrong outcomes
Hoque	2017	Understanding factors influencing the adoption of mHealth by the elderly: An extension of the UTAUT model	International Journal of Medical Informatics	Ineligible patient population
Hudson	2015	Cares (changing and advancing risk factor control through educations after stroke): A pilot trial of a transitions in care post-discharge telephone intervention for stroke patients	Neurology. Conference: 67th American Academy of Neurology Annual Meeting, AAN	Protocol
Huffman	2017	Improving blood pressure control and health systems with community health workers	JAMA - Journal of the American Medical Association	No original data
Hussain	2014	The project red chip (reducing disparities and controlling hypertension in primary care) care management intervention: An evaluation of its effectiveness & implementation	Journal of General Internal Medicine	Wrong intervention
Ionov	2021	Value-based approach to blood pressure telemonitoring and remote counseling in hypertensive patients	Blood Pressure	Wrong study design
Irewall	2019	Nurse-led, telephone-based secondary preventive follow-up benefits stroke/TIA patients with low education: a randomized controlled trial sub-study	Trials	Ineligible patient population
Islam	2019	Validation and Acceptability of a Cuffless Wrist-Worn Wearable Blood Pressure Monitoring Device Among Users and Health Care Professionals: Mixed Methods Study	JMIR Mhealth Uhealth	Wrong study design
Jackson	2012	Racial differences in the effect of a telephone-delivered hypertension disease management program	Journal of General Internal Medicine	No original data
James	2017	Participation of African Americans in e-Health and m-Health studies: a systematic review	Telemedicine and E-Health	No original data

© 2024 Katz ME et al. *JAMA Network Open*.

Jenkins	2016	Stroke patients and their attitudes toward mHealth monitoring to support blood pressure control and medication adherence	Began with 2015	Wrong outcomes
Jeong	2018	Telephone Support and Telemonitoring for Low-Income Older Adults	Research in gerontological nursing	Ineligible patient population
Jhamb	2015	Disparities in Electronic Health Record Patient Portal Use in Nephrology Clinics	Clinical Journal of The American Society of Nephrology: CJASN	Wrong study design
Jindal	2018	Development of mWellcare: an mHealth intervention for integrated management of hypertension and diabetes in low-resource settings	Global health action	Protocol
Jones	2018	Using text messages to promote health in African-Americans: #HeartHealthyandCancerFree	Ethn Health	Wrong study design
Kanaya	2012	The Live Well, Be Well study: a community-based, translational lifestyle program to lower diabetes risk factors in ethnic minority and lower-socioeconomic status adults	Am J Public Health	Wrong intervention
Kaplan	2017	Improving patient engagement in self-measured blood pressure monitoring using a mobile health technology	Health Inf Sci Syst	Wrong comparator
Kassar	2017	Use of Telemedicine for Management of Diabetes in Correctional Facilities	Telemedicine Journal & E-Health	Wrong study design
Katzmarzyk	2018	Promoting Successful Weight Loss in Primary Care in Louisiana (PROPEL): Rationale, design and baseline characteristics	Contemp Clin Trials	Protocol
Keegan	2020	Evaluating the Impact of Telehealth-Based, Diabetes Medication Training for Community Health Workers on Glycemic Control	J Pers Med	Insufficient data
Kerby	2011	Adherence to comprehensive interventions for management of uncontrolled hypertension	Journal of Clinical Hypertension	Insufficient data
Keyserling	2016	A community-based lifestyle and weight loss intervention promoting a Mediterranean-style diet pattern evaluated in the stroke belt of North Carolina: the Heart Healthy Lenoir Project	BMC Public Health	Wrong intervention
Kim	2023	The ACTIVATE Digital Health Pilot Program for Diabetes and Hypertension in an Underserved and Rural Community	Applied clinical informatics	Wrong study design
Kitsiou	2017	Development of an innovative mHealth platform for remote physical activity monitoring and health coaching of cardiac rehabilitation patients	2017 IEEE EMBS International Conference on Biomedical & Health Informatics (BHI)	Wrong outcomes
Kothapalli	2012	Factors affecting frequency of patient use of internet-based telemedicine system to manage chronic cardiovascular disease risk conditions	Journal of the American College of Cardiology	Wrong study design
Kothapalli	2013	Factors affecting frequency of patient use of Internet-based telemedicine to manage cardiovascular disease risk	Journal of Telemedicine & Telecare	Wrong outcomes
Kumar	2015	A content analysis of smartphone-based applications for hypertension management	Journal of the American Society of Hypertension	Wrong outcomes
Landry	2013	African American community members sustain favorable blood pressure outcomes through 12-month telephone motivational interviewing (MI) maintenance	FASEB Journal. Conference: Experimental Biology	Insufficient data

© 2024 Katz ME et al. *JAMA Network Open*.

Lee	2022	Early Impact of Collaborative Remote Patient Monitoring (C-RPM) for Hypertension on Underserved Populations during the COVID19 Pandemic: A Multicenter Retrospective Observational Study	Journal of the American College of Clinical Pharmacy	Insufficient data
Lee	2016	Using mHealth in Social Work Practice with Low-Income Hispanic Patients	Health & social work	No original data
Lefler	2018	Feasibility and engagement with m-health monitoring in rural-dwelling older adults with heart failure: a pilot study	Circulation	Wrong outcomes
Lewinski	2019	Addressing Diabetes and Poorly Controlled Hypertension: Pragmatic mHealth Self-Management Intervention	Journal of Medical Internet Research	Wrong study design
Li	2015	Pilot Test of a Culturally Sensitive Hypertension Management Intervention Protocol for Older Chinese Immigrants Chinese Medicine as Longevity Modality	Cin-Computers Informatics Nursing	Wrong outcomes
Lin	2020	The clinical outcomes and effectiveness of mHealth interventions for diabetes and hypertension: a systematic review and meta-analysis	medRxiv	Ineligible patient population
Liyange-Don	2023	Association between Frequency of Home Blood Pressure Measurement and Subsequent Blood Pressure Outcomes among Patients Enrolled in a Remote Patient Monitoring Program for Hypertension	Journal of General Internal Medicine	Insufficient data
Logan	2013	Transforming hypertension management using mobile health technology for telemonitoring and self-care support	Canadian Journal of Cardiology	No original data
Lokker	2021	Feasibility of a Web-Based Platform (Trial My App) to Efficiently Conduct Randomized Controlled Trials of mHealth Apps For Patients With Cardiovascular Risk Factors: Protocol For Evaluating an mHealth App for Hypertension	JMIR Research Protocols	Protocol
Lu	2019	Interactive mobile health intervention and blood pressure management in adults: a meta-analysis of randomized controlled trials	Hypertension	No original data
Lynch	2014	A self-management intervention for African Americans with comorbid diabetes and hypertension: a pilot randomized controlled trial	Prev Chronic Dis	Wrong intervention
Maciejewski	2014	Do the benefits of participation in a hypertension self-management trial persist after patients resume usual care?	Circulation. Cardiovascular Quality & Outcomes	No original data
Madsen	2008	Health-related quality of life (SF-36) during telemonitoring of home blood pressure in hypertensive patients: A randomized, controlled study	Blood Pressure	Ineligible patient population
Mainsbridge	2018	Blood Pressure Response to Interrupting Workplace Sitting Time With Non-Exercise Physical Activity: Results of a 12-Month Cohort Study	Journal of Occupational & Environmental Medicine	Ineligible patient population
Mallow	2018	The effectiveness of mI SMART: A nurse practitioner led technology intervention for multiple chronic conditions in primary care	Int J Nurs Sci	Wrong study design
Mann	2013	Development of DASH Mobile: a mHealth lifestyle change intervention for the management of hypertension	Studies in Health Technology and Informatics	Insufficient data
Mann	2014	Dietary approaches to stop hypertension: lessons learned from a case study on the development of an mHealth behavior change system	JMIR mHealth and uHealth	No original data
Marcolino	2018	The Impact of mHealth Interventions: Systematic Review of Systematic Reviews	JMIR mHealth and uHealth	No original data

© 2024 Katz ME et al. *JAMA Network Open*.

Margolis	2022	Comparing Pharmacist-Led Telehealth Care and Clinic-Based Care for Uncontrolled High Blood Pressure: The Hyperlink 3 Pragmatic Cluster-Randomized Trial	Hypertension	No original data
Margolis	2020	Cardiovascular Events and Costs With Home Blood Pressure Telemonitoring and Pharmacist Management for Uncontrolled Hypertension	Hypertension	Wrong outcomes
Martin	2011	Medication adherence among rural, low-income hypertensive adults: a randomized trial of a multimedia community-based intervention	American Journal of Health Promotion	Wrong outcomes
McCarroll	2017	Effectiveness of mobile health (mHealth) interventions for promoting healthy eating in adults: A systematic review	Preventive Medicine	No original data
McGillicuddy	2020	Exploratory analysis of the impact of an mhealth medication adherence intervention on tacrolimus trough concentration variability: post hoc results of a randomized controlled trial	Annals of Pharmacotherapy	Wrong outcomes
McLaughlin	2010	Heart failure: Telephone monitoring improves outcomes in patients with CHF	Nature Reviews Cardiology	No original data
Melville	2015	Next-generation cloud-based blood pressure devices in chronic disease management: A direct intraarterial pressure calibration of an oscillometric wrist cuff device for clinically reliable and accurate blood pressure measurements	Canadian Journal of Cardiology	Wrong outcomes
Memon	2010	Patient centered care: Baseline demographics data from a stage I hypertension clinical trial	Circulation: Cardiovascular Quality and Outcomes. Conference: Quality of Care and Outcomes Research in Cardiovascular Disease and Stroke	Wrong outcomes
Mertens	2017	Mobile Technology Improves Therapy-Adherence Rates in Elderly Patients Undergoing Rehabilitation-A Crossover Design Study	Advances in Human Factors and Ergonomics in Healthcare	Wrong outcomes
Meurer	2020	Reach out behavioral intervention for hypertension initiated in the emergency department connecting multiple health systems: study protocol for a randomized control trial	Trials [Electronic Resource]	Protocol
Michalakeas	2020	Mobile phones and applications in the management of patients with arterial hypertension	American Journal of Cardiovascular Disease	No original data
Milani	2016	The Role of Technology in Chronic Disease Care	Progress in Cardiovascular Diseases	No original data
Mirsky	2020	Chronic disease management in the COVID-19 era	American Journal of Managed Care	No original data
Misher	2020	Employer-Sponsored Wellness Programs for Hypertension and Dyslipidemia in a 2-Hospital Health System	Am Health Drug Benefits	Wrong intervention
Morawski	2017	Rationale and design of the Medication adherence Improvement Support App For Engagement-Blood Pressure (MedISAFE-BP) trial	American Heart Journal	Protocol
Nct	2016	mHealth for Diabetes Adherence Support	Clinicaltrials.gov	Protocol
Nct	2019	A Pharmacist Intervention for Monitoring and Treating Hypertension Using Bidirectional Texting	Clinicaltrials.gov	Protocol
Nelson	2014	Peer Support for Achieving Independence in Diabetes (Peer-AID): design, methods and baseline characteristics of a randomized controlled trial of community health worker assisted diabetes self-management support	Contemp Clin Trials	Protocol

© 2024 Katz ME et al. *JAMA Network Open*.

Nicklas	2016	Participant feedback and iterative improvements to a mobile health program for postpartum women at elevated cardiometabolic risk	Journal of General Internal Medicine	Wrong outcomes
NnodimOpara	2023	Linkage, Empowerment, and Access to Prevent Hypertension: A Novel Program to Prevent Hypertension and Reduce Cardiovascular Health Disparities in Detroit, Michigan	American Journal of Hypertension	No original data
Oclaman	2023	The Association Between Mobile App Use and Change in Functional Capacity Among Cardiac Rehabilitation Participants: Cohort Study	JMIR Cardiology	Wrong comparator
Oh	2022	Effect of an Integrative Mobile Health Intervention in Patients With Hypertension and Diabetes: Crossover Study	JMIR MHealth UHealth	Ineligible patient population
Olfert	2019	Feasibility of a mHealth Approach to Nutrition Counseling in an Appalachian State	Journal of Personalized Medicine	Wrong outcomes
Omboni	2019	Connected health in hypertension management	Frontiers in cardiovascular medicine	No original data
Or	2016	A 3-Month Randomized Controlled Pilot Trial of a Patient-Centered, Computer-Based Self-Monitoring System for the Care of Type 2 Diabetes Mellitus and Hypertension	Journal of Medical Systems	Ineligible patient population
Otero-Sabogal	2011	Physician-community health worker partnering to support diabetes self-management in primary care	Qual Prim Care	Wrong intervention
Padwal	2019	Designing interventions for blood pressure control in challenging settings: Active not passive intervention is needed	Journal of Clinical Hypertension	No original data
Palmas	2008	Telemedicine home blood pressure measurements and progression of albuminuria in elderly people with diabetes	Hypertension	Wrong outcomes
Palmas	2014	Results of the northern Manhattan diabetes community outreach project: a randomized trial studying a community health worker intervention to improve diabetes care in Hispanic adults	Diabetes Care	Wrong intervention
Parati	2009	Home blood pressure telemonitoring improves hypertension control in general practice. the TeleBPCare study	Journal of hypertension	Ineligible patient population
Pare	2007	Systematic review of home telemonitoring for chronic diseases: the evidence base	Journal of the American Medical Informatics Association	No original data
Parola	2022	Assessing Patient Engagement and Participation in Remote Patient Monitoring Program for Hypertension	Journal of General Internal Medicine	Insufficient data
Pemu	2019	Achieving Health Equity with e-Healthystrides: Patient Perspectives of a Consumer Health Information Technology Application	Ethnicity & disease	Wrong study design
Perdigao	2020	Postpartum blood pressure trends are impacted by race and BMI	Pregnancy Hypertension-an International Journal of Womens Cardiovascular Health	Insufficient data
Peters	2017	Assessing the Utility of a Novel SMS- and Phone-Based System for Blood Pressure Control in Hypertensive Patients: Feasibility Study	JMIR Cardio	Wrong study design

© 2024 Katz ME et al. *JAMA Network Open*.

Piette	2011	Clinical complexity and the effectiveness of an intervention for depressed diabetes patients	Chronic Illn	Wrong intervention
Piette	2015	Mobile Health Devices as Tools for Worldwide Cardiovascular Risk Reduction and Disease Management	Circulation	No original data
Pletcher	2020	The PCORnet Blood Pressure Control Laboratory: A Platform for Surveillance and Efficient Trials	Circulation. Cardiovascular Quality & Outcomes	No original data
Poblete	2023	Digitally Based Blood Pressure Self-Monitoring Program That Promotes Hypertension Self-Management and Health Education Among Patients With Low-Income: Usability Study	JMIR Human Factors	Wrong comparator
Post	2013	New media use by patients who are homeless: the potential of mHealth to build connectivity	Journal of Medical Internet Research	Wrong outcomes
Prabhakaran	2019	Effectiveness of an mHealth-based electronic decision support system for integrated management of chronic conditions in primary care: the mWellcare cluster-randomized controlled trial	Circulation	Ineligible patient population
Prendergast	2020	Preliminary Data from a Randomized Controlled Trial for a Hypertension Education and Empowerment Intervention (TOUCHED) in an Urban, Academic Emergency Department: Opportunities in the era of COVID-19	Journal of the National Medical Association	Protocol
Raber	2019	Health Insurance and Mobile Health Devices: Opportunities and Concerns	JAMA - Journal of the American Medical Association	No original data
Ramirez	2016	Assessing the Use of Mobile Health Technology by Patients: An Observational Study in Primary Care Clinics	JMIR mHealth and uHealth	Wrong outcomes
Randall	2019	The effects of telehealth on the frequency of primary care clinic visits	Journal of General Internal Medicine	Wrong outcomes
Rehman	2017	Mobile Health (mHealth) Technology for the Management of Hypertension and Hyperlipidemia: Slow Start but Loads of Potential	Current Atherosclerosis Reports	No original data
Reidpath	2012	Community-based blood pressure measurement by non-health workers using electronic devices: a validation study	Glob Health Action	Ineligible patient population
Rhoads	2017	Exploring implementation of m-health monitoring in postpartum women with hypertension	Telemedicine and E-Health	Wrong outcomes
Rich	2017	Implementation-effectiveness trial of an ecological intervention for physical activity in ethnically diverse low income senior centers	BMC Public Health	Protocol
Rivera-Romero	2018	Mobile Health Solutions for Hypertensive Disorders in Pregnancy: Scoping Literature Review	JMIR mHealth and uHealth	No original data
Roess	2017	The promise, growth, and reality of mobile health - Another data-free zone	New England Journal of Medicine	No original data
Siafi	2022	Blood Pressure Management with the Esh Care App - Preliminary Results	Journal of hypertension	Ineligible patient population

© 2024 Katz ME et al. *JAMA Network Open*.

Salter	2014	Implementation of telecommunication stroke rehabilitation within stroke early supported discharge team (ESD)	International Journal of Stroke	Ineligible patient population
Samuel-Hodge	2013	A pilot study comparing two weight loss maintenance interventions among low-income, mid-life women	BMC Public Health	Wrong intervention
Samuel-Hodge	2020	Strengthening community-clinical linkages to reduce cardiovascular disease risk in rural NC: feasibility phase of the CHANGE study	BMC Public Health	Wrong intervention
Saner	2019	Digital health implementation: How to overcome the barriers?	European Journal of Preventive Cardiology	No original data
Santo	2019	The potential of mHealth applications in improving resistant hypertension self-assessment, treatment and control	Current Hypertension Reports	No original data
Schoenthaler	2020	Development and Evaluation of a Tailored Mobile Health Intervention to Improve Medication Adherence in Black Patients With Uncontrolled Hypertension and Type 2 Diabetes: Pilot Randomized Feasibility Trial	JMIR mHealth and uHealth	Insufficient data
Senecal	2018	Usage of a Digital Health Workplace Intervention Based on Socioeconomic Environment and Race: Retrospective Secondary Cross-Sectional Study	J Med Internet Res	Protocol
Shah	2015	Telemonitoring of blood pressure in low-income African American patients with congestive heart failure	Hypertension. Conference: American Heart Association's Council on Hypertension	Insufficient data
Shah	2021	Identifying features of successful mobile health interventions for hypertension self-management in populations with digital barriers: A qualitative comparative analysis	Journal of General Internal Medicine	No original data
Shaw	2013	Development of a theoretically driven mHealth text messaging application for sustaining recent weight loss	JMIR mHealth and uHealth	Wrong outcomes
Shea	2006	A randomized trial comparing telemedicine case management with usual care in older, ethnically diverse, medically underserved patients with diabetes mellitus	Journal of the American Medical Informatics Association	No original data
Shea	2007	The Informatics for Diabetes and Education Telemedicine (IDEATel) project	Transactions of the American Clinical and Climatological Association	No original data
Shea	2013	Social impact analysis of the effects of a telemedicine intervention to improve diabetes outcomes in an ethnically diverse, medically underserved population: findings from the IDEATel Study	Am J Public Health	Insufficient data
Sieverdes	2013	mHealth medication and blood pressure self-management program in Hispanic hypertensives: a proof of concept trial	Smart Homecare Technology and TeleHealth	Insufficient data
Sieverdes	2017	Formative evaluation on cultural tailoring breathing awareness meditation smartphone apps to reduce stress and blood pressure	Began with 2015	Wrong study design
Singh-Franco	2013	Improvement in surrogate endpoints by a multidisciplinary team in a mobile clinic serving a low-income, immigrant minority population in South Florida	Journal of Health Care for the Poor & Underserved	Wrong intervention
Skolarus	2023	Reach Out Emergency Department: A Randomized Factorial Trial to Determine the Optimal Mobile Health Components to Reduce Blood Pressure	Circulation Cardiovascular Quality and Outcomes	Wrong study design

© 2024 Katz ME et al. *JAMA Network Open*.

Song	2013	Mobile clinic in Massachusetts associated with cost savings from lowering blood pressure and emergency department use	Health Affairs	Wrong intervention
Spiegelman	2020	430: Remote postpartum blood pressure surveillance for hypertensive disorders of pregnancy: a randomized clinical trial	American Journal of Obstetrics and Gynecology	Wrong outcomes
Spring	2018	Multicomponent mHealth intervention for large, sustained change in multiple diet and activity risk behaviors: the make better choices 2 randomized controlled trial	Journal of Medical Internet Research	Wrong outcomes
Spruill	2015	Comparative effectiveness of home blood pressure telemonitoring (HBPTM) plus nurse case management versus HBPTM alone among Black and Hispanic stroke survivors: study protocol for a randomized controlled trial	Trials [Electronic Resource]	Protocol
Steinberg	2019	The Effect of a Digital Behavioral Weight Loss Intervention on Adherence to the Dietary Approaches to Stop Hypertension (DASH) Dietary Pattern in Medically Vulnerable Primary Care Patients: Results from a Randomized Controlled Trial	J Acad Nutr Diet	Wrong intervention
Steinhubl	2015	The emerging field of mobile health	Science translational medicine	No original data
Taber	2018	Pharmacist-led, technology-assisted study to improve medication safety, cardiovascular risk factor control, and racial disparities in kidney transplant recipients	JACCP Journal of the American College of Clinical Pharmacy	Wrong study design
Taylor	2020	Preventing Cardiovascular Disease Among Urban African Americans With a Mobile Health App (the MOYO App): Protocol for a Usability Study	JMIR Research Protocols	Protocol
Thangada	2018	The emerging role of mobile-health applications in the management of hypertension	Current Cardiology Reports	No original data
Thomas	2016	Mobile Health Technology Can Objectively Capture Physical Activity (PA) Targets Among African-American Women Within Resource-Limited Communities-the Washington, D.C. Cardiovascular Health and Needs Assessment	J Racial Ethn Health Disparities	Wrong intervention
Threatt	2017	Telehealth for diabetes self-management education and support in an underserved, free clinic population: A pilot study	Journal of the American Pharmacists Association	Insufficient data
Towfighi	2021	Effect of a Coordinated Community and Chronic Care Model Team Intervention vs Usual Care on Systolic Blood Pressure in Patients With Stroke or Transient Ischemic Attack: The SUCCEED Randomized Clinical Trial	JAMA Netw Open	Wrong intervention
Tuot	2015	The Kidney Awareness Registry and Education (KARE) study: protocol of a randomized controlled trial to enhance provider and patient engagement with chronic kidney disease	BMC Nephrology	Protocol
Turner	2021	Sharing patient-generated data with healthcare providers: findings from a 2019 national survey	Journal of the American Medical Informatics Association	Wrong outcomes
Ueda	2017	Effects of a home blood pressure monitoring by mobile phone-based self management support system in mild hypertension: the Katsuragi study	Journal of Hypertension	Ineligible patient population
Ueda	2020	Effects of a home blood pressure monitoring by mobile phone-based and health service (continuous antihypertensive treatment) in mild hypertension : the wakayama health promotion study	Atherosclerosis	Ineligible patient population

© 2024 Katz ME et al. *JAMA Network Open*.

Val Overton	2021	Mobile Health Intervention to Close the Guidelines-To-Practice Gap in Hypertension Treatment: Protocol for the mGlide Randomized Controlled Trial		Protocol
Vandenberk	2019	Relationship between adherence to remote monitoring and patient characteristics: observational study in women with pregnancy-induced hypertension	JMIR mHealth and uHealth	Ineligible patient population
Vasti	2020	Recruiting Student Health Coaches to Improve Digital Blood Pressure Management: Randomized Controlled Pilot Study	JMIR Form Res	Wrong comparator
Vaughan	2023	Implementation and Evaluation of a mHealth-Based Community Health Worker Feedback Loop for Hispanics with and at Risk for Diabetes	Journal of General Internal Medicine	No original data
Verdejo	2015	Heart Failure in Rural Communities	Heart Failure Clinics	No original data
Vilme	2019	Using Telehealth to Disseminate Primary, Secondary, and Tertiary CVD Interventions to Rural Populations	Current Hypertension Reports	No original data
Vo	2019	Patients perceptions of mHealth apps: meta-ethnographic review of qualitative studies	JMIR mHealth and uHealth	No original data
Wang	2017	A systematic review of application and effectiveness of mHealth interventions for obesity and diabetes treatment and self-management	Advances in Nutrition	No original data
Wang	2021	Telemedicine in the management of hypertension: Evolving technological platforms for blood pressure telemonitoring	Journal of Clinical Hypertension	No original data
Weerahandi	2020	A Mobile Health Coaching Intervention for Controlling Hypertension: Single-Arm Pilot Pre-Post Study	JMIR Form Res	Wrong study design
Welsh	2016	Strategies in Development of an mHealth Technology for Low Socioeconomic Groups in Free Healthcare Clinics	CIN: Computers, Informatics, Nursing	Wrong intervention
Whit	2020	Reach Out: Recruitment in a multicomponent, mobile health, behavioral intervention to reduce blood pressure in the Emergency Department patient population	Journal of Stroke and Cerebrovascular Diseases	Protocol
White	2011	Home blood pressure monitoring as an intervention to control hypertension	Archives of Internal Medicine	No original data
Whitfield	2020	Adapting reach out: A mobile health intervention trial, in response to the coronavirus pandemic	Annals of Neurology	Wrong outcomes
Wilber	1972	Hypertension--a community problem	American Journal of Medicine	Wrong intervention
Xiong	2018	Effectiveness of mHealth Interventions in Improving Medication Adherence Among People with Hypertension: a Systematic Review	Current Hypertension Reports	No original data
Yun	2020	Efficacy of an electronic health management program for patients with cardiovascular risk: randomized controlled trial	Journal of Medical Internet Research	Ineligible patient population
Zanowiak	2019	A community health worker-led health education and coaching intervention improves hypertension control among south Asian primary care patients in New York city	Circulation. Conference: American Heart Association Scientific Sessions, AHA	Wrong intervention

© 2024 Katz ME et al. *JAMA Network Open*.

Zheng	2020	One-year Patterns of Home Blood Pressure Monitoring Using Consumer-purchased Wireless Devices in the Health Eheart Study	Circulation. Conference: American Heart Association Scientific Sessions, AHA	Wrong study design
Zoellner	2011	H.U.B city steps: Methods and early findings from a community-based participatory research trial to reduce blood pressure among african americans	International Journal of Behavioral Nutrition and Physical Activity	Wrong intervention
Zullig	2015	Patient-reported medication adherence barriers among veterans affairs patients with cardiovascular risk factors	Journal of General Internal Medicine	Wrong intervention
Zullig	2018	Changing CHANGE: adaptations of an evidence-based telehealth cardiovascular disease risk reduction intervention	Translational Behavioral Medicine	Wrong outcomes

© 2024 Katz ME et al. *JAMA Network Open*.

eTable 5. Excluded studies that were eliminated from the Citation Chasing with corresponding rationale

First Authors Last Name	Year	Title	Journal	Reason for Exclusion
Baig	2015	Picture Good Health: A Church-Based Self-Management Intervention Among Latino Adults with Diabetes	Journal of general internal medicine	Wrong intervention
Bobrow	2016	Mobile Phone Text Messages to Support Treatment Adherence in Adults with High Blood Pressure (SMS-Text Adherence Support [StAR]): A Single-Blind, Randomized Trial	Circulation	Ineligible patient population
Bosworth	2011	Home blood pressure management and improved blood pressure control: results from a randomized controlled trial	Archives of internal medicine	No original data
Bove	2011	Reducing cardiovascular disease risk in medically underserved urban and rural communities	American heart journal	No original data
Buis	2020	Understanding the Feasibility, Acceptability, and Efficacy of a Clinical Pharmacist-led Mobile Approach (BPTrack) to Hypertension Management: Mixed Methods Pilot Study	Journal of medical Internet research	Insufficient Data
Carter	2015	Cluster-Randomized Trial of a Physician/Pharmacist Collaborative Model to Improve Blood Pressure Control	Circulation. Cardiovascular quality and outcomes	Wrong intervention
Cena	2016	A multicomponent quality improvement intervention to improve blood pressure and reduce racial disparities in rural primary care practices	Journal of clinical hypertension	Wrong study design
Chow	2015	Effect of Lifestyle-Focused Text Messaging on Risk Factor Modification in Patients With Coronary Heart Disease: A Randomized Clinical Trial	JAMA	Ineligible patient population
Collins	2019	Efficacy of a multi-component intervention to promote physical activity among Latino adults: A randomized controlled trial	Preventive medicine reports	Insufficient data
Contreras	2018	Specific hypertension smartphone application to improve medication adherence in hypertension: a cluster-randomized trial	Current medical research and opinion	Ineligible patient population
Davidson	2015	Evaluation of an mHealth Medication Regimen Self-Management Program for African American and Hispanic Uncontrolled Hypertensives	Journal of personalized medicine	Insufficient data
Ewald	2006	Relationship between the frequency of blood pressure self-measurement and blood pressure reduction with antihypertensive therapy : results of the OLMETEL (OLMEsartan TELemonitoring blood pressure) study	Clinical drug investigation	Ineligible patient population
Field	2002	Telemedicine and remote patient monitoring	JAMA	Insufficient data
Frie	2020	Effectiveness of a self-regulation intervention for weight loss: A randomized controlled trial	British journal of health psychology	Ineligible patient population
Friedman	1996	A Telecommunications System for Monitoring and Counseling Patients with Hypertension: Impact on Medication Adherence and Blood Pressure Control	American Journal of Hypertension	Wrong intervention
Gonzalez-Sanchez	2019	Using a smartphone app in changing cardiovascular risk factors: A randomized controlled trial (EVIDENT II study)	International journal of medical informatics	Ineligible patient population

© 2024 Katz ME et al. *JAMA Network Open*.

Green	2008	Effectiveness of Home Blood Pressure Monitoring, Web Communication, and Pharmacist Care on Hypertension Control: A Randomized Controlled Trial	JAMA	Ineligible patient population
Hachsmann	2021	Effects of a 2-Year Primary Care Lifestyle Intervention on Cardiometabolic Risk Factors: A Cluster-Randomized Trial	Circulation	Wrong intervention
Hicks	2008	Impact of Computerized Decision Support on Blood Pressure Management and Control: A Randomized Controlled Trial	Journal of general internal medicine	Wrong outcomes
Kes	2021	The effect of nurse-led telephone support on adherence to blood pressure control and drug treatment in individuals with primary hypertension: A randomized controlled study	International journal of nursing practice	Ineligible patient population
Kumanyika	2018	Two-Year Results of Think Health! Vive Saludable!: A Primary Care Weight-Management Trial	Obesity	Wrong intervention
Liang	2015	Behavioral support intervention for uncontrolled hypertension: a complier average causal effect (CACE) analysis	Medical care	Wrong outcomes
Logan	2012	Effect of Home Blood Pressure Telemonitoring With Self-Care Support on Uncontrolled Systolic Hypertension in Diabetics	Hypertension	Wrong study design
Magid	2011	A multimodal blood pressure control intervention in 3 healthcare systems	The American journal of managed care	Insufficient data
McGillicuddy	2015	Sustainability of improvements in medication adherence through a mobile health intervention	Progress in transplantation	Insufficient data
McManus	2010	Telemonitoring and self-management in the control of hypertension (TASMINH2): a randomised controlled trial	Lancet (London, England)	Ineligible patient population
McManus	2021	Home and Online Management and Evaluation of Blood Pressure (HOME BP) using a digital intervention in poorly controlled hypertension: randomised controlled trial	BMJ (Clinical research ed.)	Ineligible patient population
Mehta	2019	Electronic Pill Bottles or Bidirectional Text Messaging to Improve Hypertension Medication Adherence (Way 2 Text): a Randomized Clinical Trial	Journal of general internal medicine	Wrong intervention
Park	2021	Adherence to Telemonitoring Therapy for Medicaid Patients With Hypertension: Case Study	Journal of medical Internet research	Wrong study design
Philis-Tsimikas	2014	Dulce Mothers: an intervention to reduce diabetes and cardiovascular risk in Latinas after gestational diabetes	Translational behavioral medicine	Wrong intervention
Rogers	2001	Home Monitoring Service Improves Mean Arterial Pressure in Patients with Essential Hypertension: A Randomized, Controlled Trial	Annals of internal medicine	Insufficient data
Santamore	2007	Using a Telemedicine System to Decrease Cardiovascular Disease Risk in an Underserved Population: Design, Use, and Interim Results	Annual International Conference of the IEEE Engineering in Medicine and Biology Society. IEEE Engineering in Medicine and Biology Society. Annual International Conference	Insufficient data

© 2024 Katz ME et al. *JAMA Network Open*.

Schoenthaler	2019	Development and Evaluation of a Tailored Mobile Health Intervention to Improve Medication Adherence in Black Patients With Uncontrolled Hypertension and Type 2 Diabetes: Pilot Randomized Feasibility Trial (Preprint)	NA	Wrong study design
Still	2021	A Pilot Study Evaluating the Effects of a Technology-Based and Positive Psychological Training Intervention on Blood Pressure in African Americans With Hypertension	Journal of primary care & community health	Insufficient data
Volpp	2017	Effect of Electronic Reminders, Financial Incentives, and Social Support on Outcomes After Myocardial Infarction: The HeartStrong Randomized Clinical Trial	JAMA internal medicine	Wrong outcomes
Wakefield	2011	Effectiveness of Home Telehealth in Comorbid Diabetes and Hypertension: A Randomized, Controlled Trial	Telemedicine journal and e-health	Insufficient data
Yue	2021	Home blood pressure telemonitoring for improving blood pressure control in middle-aged and elderly patients with hypertension	Journal of clinical hypertension	Ineligible patient population

© 2024 Katz ME et al. *JAMA Network Open*.

eTable 6. Subgroup analysis showing change in SBP at 6 months

Subgroup	Number of studies	Heterogeneity (I^2)	Difference in change in SBP, treatment vs control	p-value subgroup comparison
**Overall, change in SBP, 6month**	**10**	**80.3%**	**-2.74 (-6.43, 0.95)**	**n/a**
Used remote BP monitoring	7	82.7%	-4.22 (-9.17, 0.72)	0.24
- no remote BP monitoring	3	0%	-0.26 (-2.95, 2.43)	
Race/ethnic focused	2	0%	3.91 (-1.60, 9.42)	0.12
- not race based	8	81.8%	-3.95 (-7.88, -0.01)	
Pilot study	2	0%	3.91 (-1.60, 9.42)	0.12
- not pilot study	8	81.8%	-3.95 (-7.88, -0.01)	
BP primary outcome	6	86.5%	-4.17 (-9.65, 1.30)	0.30
- BP not primary outcome	4	0%	-0.44 (-3.07, 2.20)	
BP controlled at baseline, ≤140	4	29.3%	0.85 (-2.00, 3.69)	0.02
- BP not controlled at baseline	6	75.7%	-5.87 (-10.55, -1.18)	

Abbreviations: SBP, systolic blood pressure; BP, blood pressure.

© 2024 Katz ME et al. *JAMA Network Open*.

eTable 7. Subgroup analysis showing change in DBP at 6 months

Subgroup	Number of studies	Heterogeneity (I^2)	Difference in change in DBP, treatment vs control	p-value subgroup comparison
**Overall, change in DBP, 6month**	**10**	**70.5%**	**-1.11 (-3.09, 0.87)**	**n/a**
Used remote BP monitoring	7	78.9%	-1.30 (-4.20, 1.61)	0.71
- no remote BP monitoring	3	0%	-0.97 (-2.71, 0.77)	
Race/ethnic focused	2	0%	1.57 (-2.59, 5.73)	0.31
- not race based	8	75.9%	-1.50 (-3.70, 0.71)	
Pilot study	2	0%	1.57 (-2.59, 5.73)	0.31
- not pilot study	8	75.9%	-1.50 (-3.70, 0.71)	
BP primary outcome	6	79.0%	-1.93 (-4.86, 0.99)	0.31
- BP not primary outcome	4	0%	-0.62 (-2.30, 1.07)	
BP controlled at baseline, ≤140	4	50.7%	0.22 (-2.05, 2.48)	0.17
- BP not controlled at baseline	6	72.1%	-2.18 (-5.02, 0.66)	
Abbreviations: DBP, systolic blood pressure; BP, blood pressure.				

© 2024 Katz ME et al. *JAMA Network Open*.

eTable 8. Subgroup analysis showing SBP values at 6 months

Subgroup	Number of studies	Heterogeneity (I^2)	Difference in SBP, treatment vs control	p-value subgroup comparison
**Overall, SBP at 6month**	**12**	**77.4%**	**-4.24 (-7.33, -1.14)**	**n/a**
Used remote BP monitoring	8	81.6%	-5.64 (-9.49, -1.79)	0.16
- no remote BP monitoring	4	21.9%	-1.04 (-4.65, 2.57)	
Race/ethnic focused	5	0%	-2.54 (-5.20, 0.12)	0.26
- not race based	7	85.5%	-5.56 (-10.04, -1.09)	
Pilot study	4	0%	-3.41 (-6.48, -0.34)	0.73
- not pilot study	8	84.9%	-4.51 (-8.66, -0.36)	
BP primary outcome	8	81.5%	-5.82 (-9.52, -2.12)	0.08
- BP not primary outcome	4	0%	0.14 (-3.28, 3.56)	
BP controlled at baseline, ≤140	6	16.0%	-1.36 (-4.11, 1.38)	0.10
- BP not controlled at baseline	6	83.7%	-6.35 (-10.79, -1.92)	
Abbreviations: SBP, systolic blood pressure; BP, blood pressure.				

© 2024 Katz ME et al. *JAMA Network Open*.

eTable 9. Subgroup analysis showing DBP values at 6 months

Subgroup	Number of studies	Heterogeneity (I^2)	Difference in DBP, treatment vs control	p-value subgroup comparison
**Overall, DBP at 6month**	**12**	**67.6%**	**-1.86 (-3.82, 0.10)**	**n/a**
Used remote BP monitoring	8	73.5%	-2.42 (-5.10, 0.26)	0.42
- no remote BP monitoring	4	21.0%	-0.51 (-2.67, 1.65)	
Race/ethnic focused	5	3.7%	-0.85 (-3.09, 1.39)	0.44
- not race based	7	80.7%	-2.46 (-5.32, 0.39)	
Pilot study	4	8.1%	-2.25 (-5.36, 0.85)	0.85
- not pilot study	8	77.8%	-1.74 (-4.19, 0.71)	
BP primary outcome	8	73.0%	-2.67 (-5.18, -0.16)	0.20
- BP not primary outcome	4	0%	0.29 (-1.73, 2.31)	
BP controlled at baseline, ≤140	6	43.2%	-0.71 (-2.84, 1.43)	0.31
- BP not controlled at baseline	6	71.8%	-2.75 (-5.75, 0.26)	
Abbreviations: DBP, diastolic blood pressure; BP, blood pressure.				

© 2024 Katz ME et al. *JAMA Network Open*.

eFigure 1. Differences in diastolic blood pressure changes from baseline to follow-up time points between digital health intervention and control groups

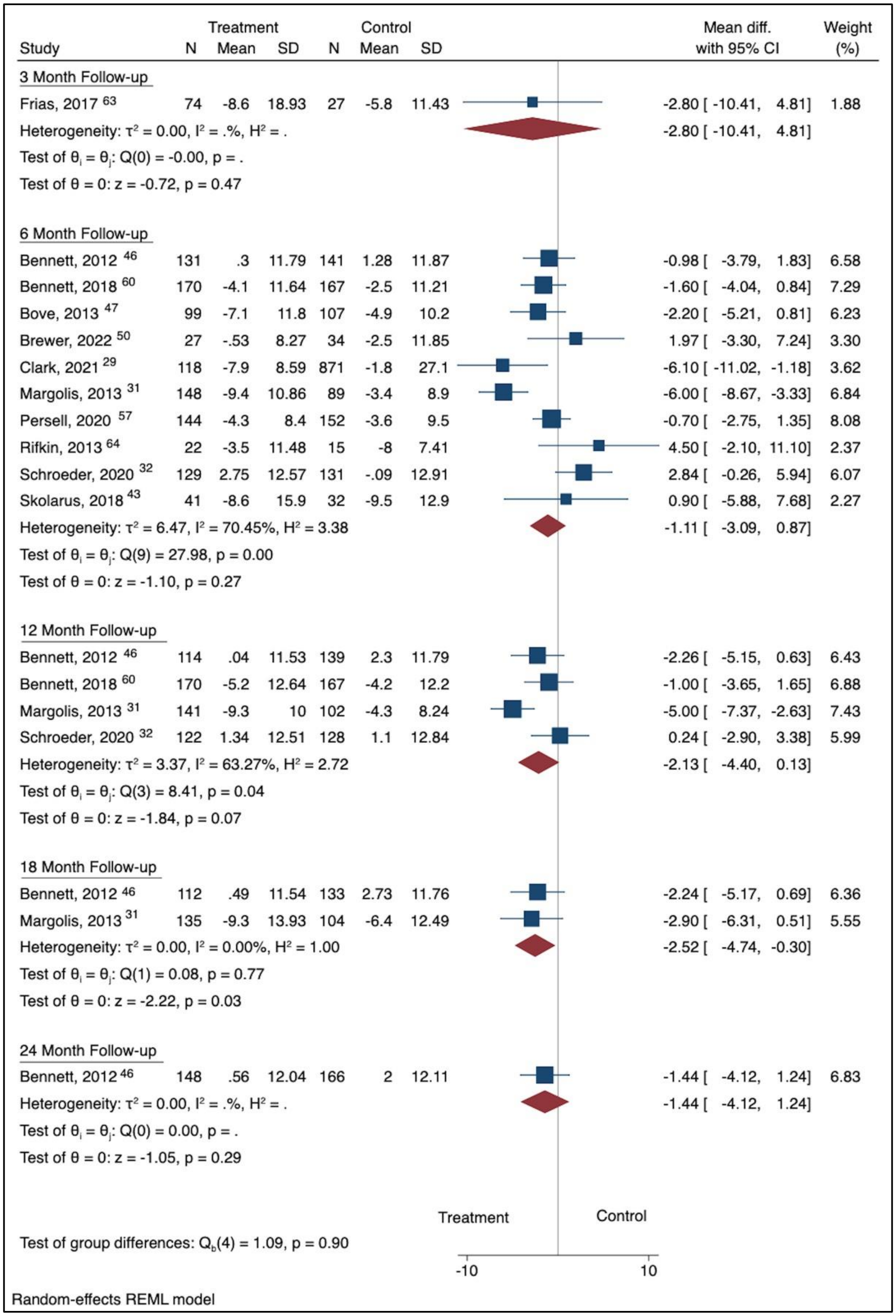

© 2024 Katz ME et al. *JAMA Network Open*.

eFigure 2. Differences in follow-up diastolic blood pressures between digital health intervention and control groups at different time points

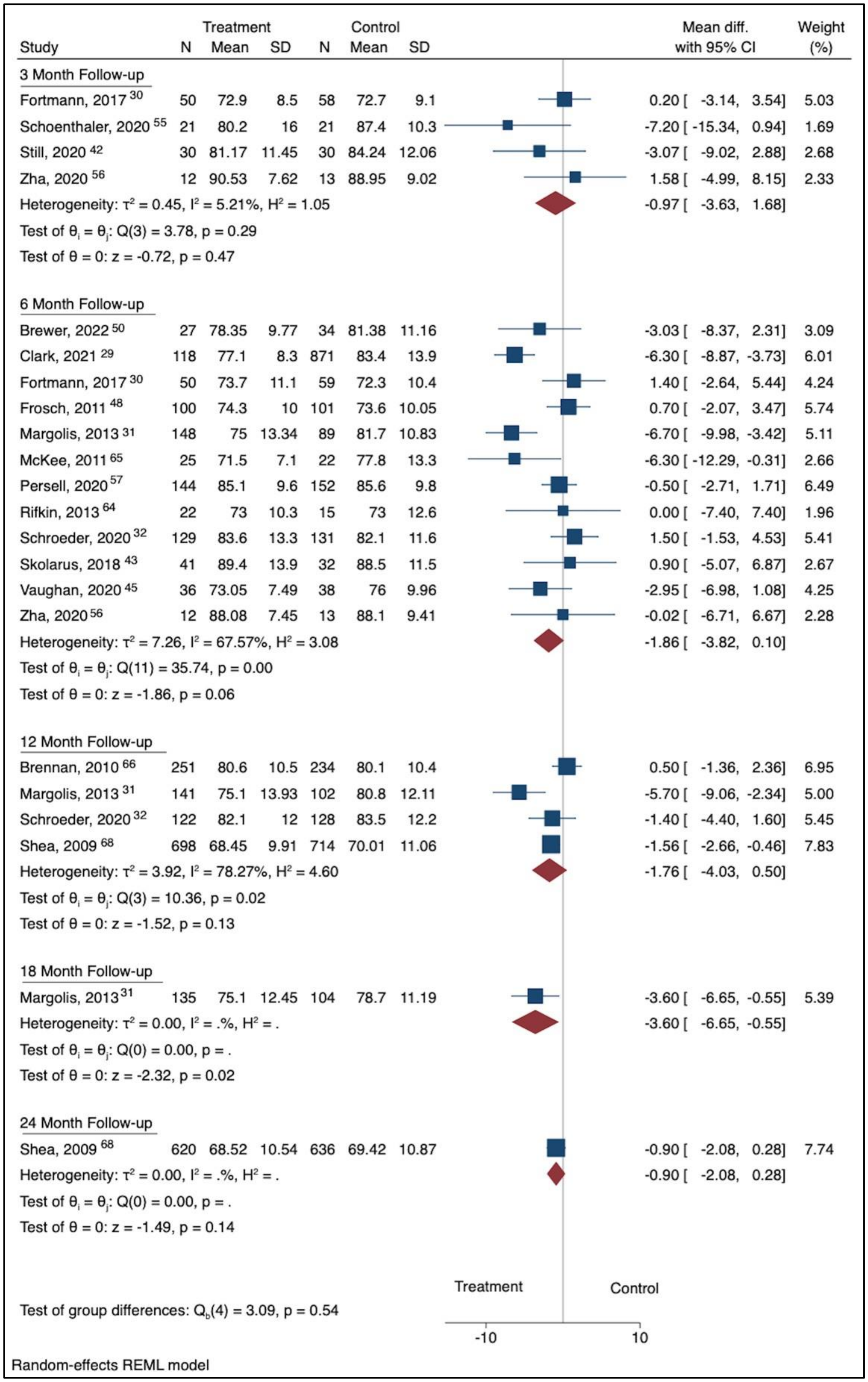

© 2024 Katz ME et al. *JAMA Network Open*.

eFigure 3. Sensitivity analysis assessing for the presence of publication bias using the change in systolic blood pressure (SBP) outcome at the 6-month follow-up duration (top); leave-one-out meta-analysis results for the same outcome as above (bottom)

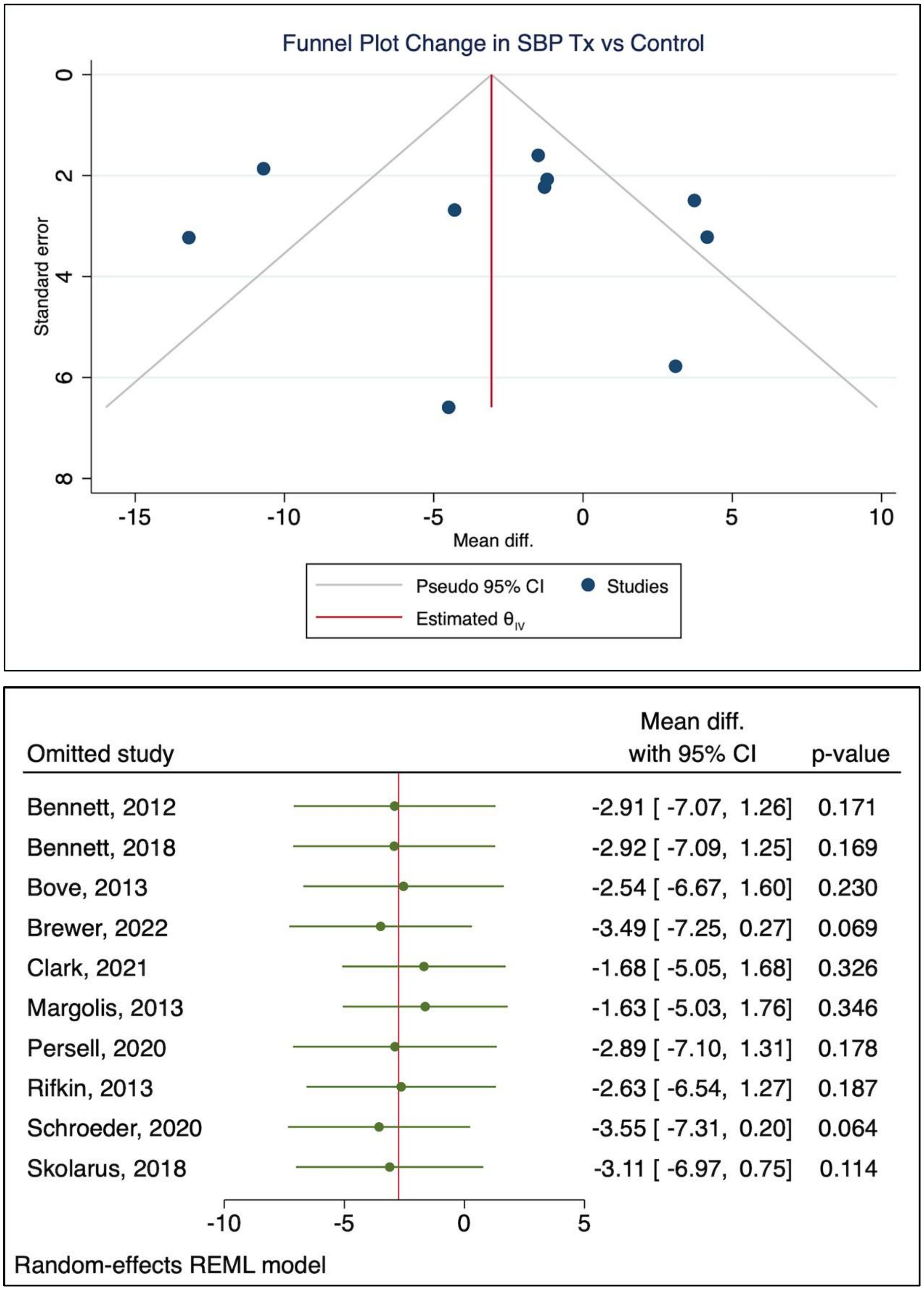

© 2024 Katz ME et al. *JAMA Network Open*.

eFigure 4. Sensitivity analysis assessing for the presence of publication bias using the follow-up systolic blood pressure (SBP) value outcome at the 6-month follow-up duration (top); leave-one-out meta-analysis results for the same outcome as above (bottom)

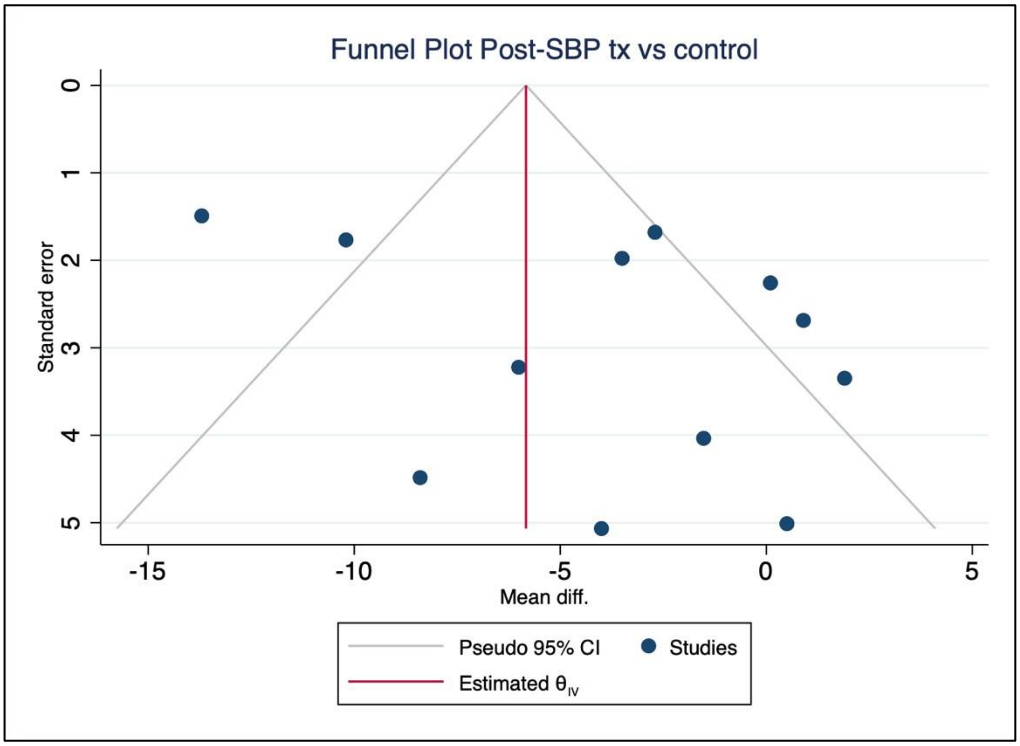

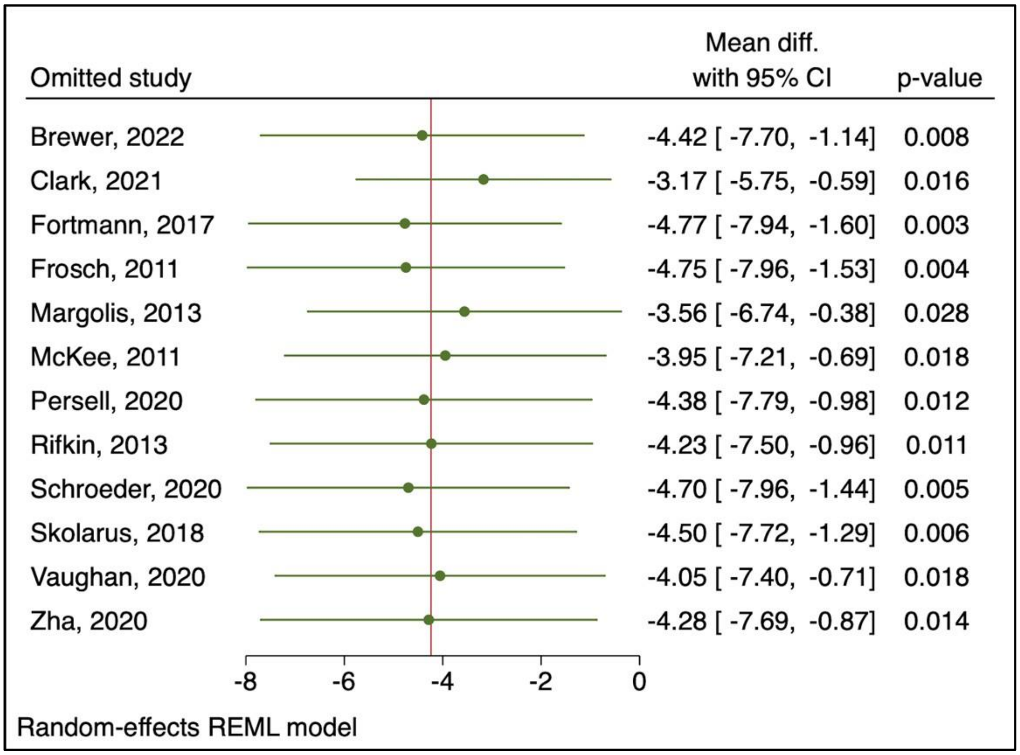

© 2024 Katz ME et al. *JAMA Network Open*.

eFigure 5. Sensitivity analysis assessing for the presence of publication bias using the change in diastolic blood pressure (DBP) outcome at the 6-month follow-up duration (top); leave-one-out meta-analysis results for the same outcome as above (bottom)

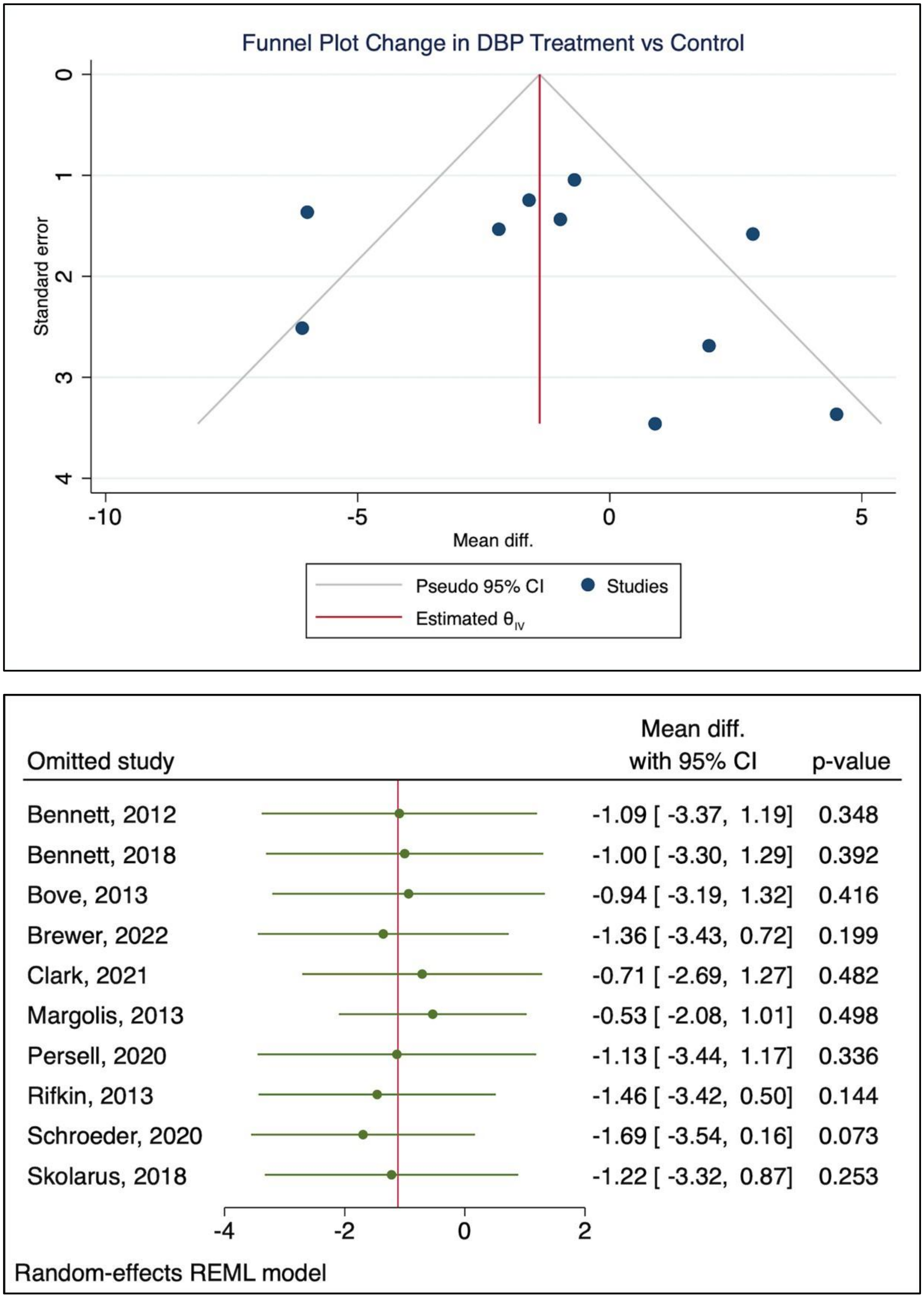

© 2024 Katz ME et al. *JAMA Network Open*.

eFigure 6. Sensitivity analysis assessing for the presence of publication bias using the follow-up diastolic blood pressure (DBP) value outcome at the 6-month follow-up duration (top); leave-one-out meta-analysis results for the same outcome as above (bottom)

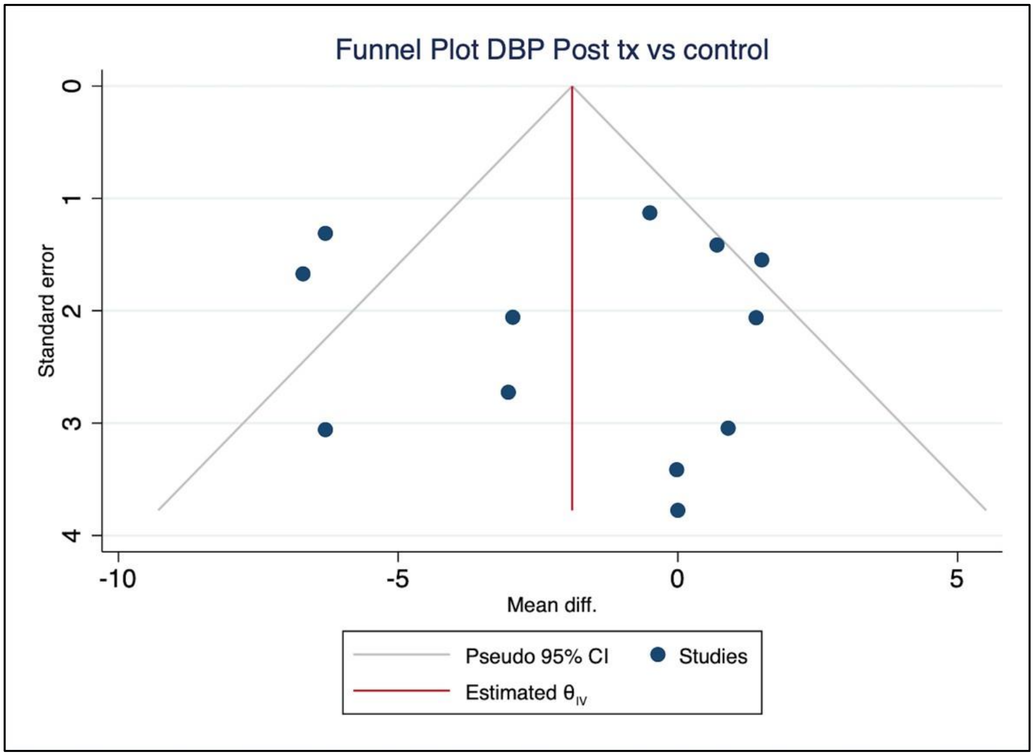

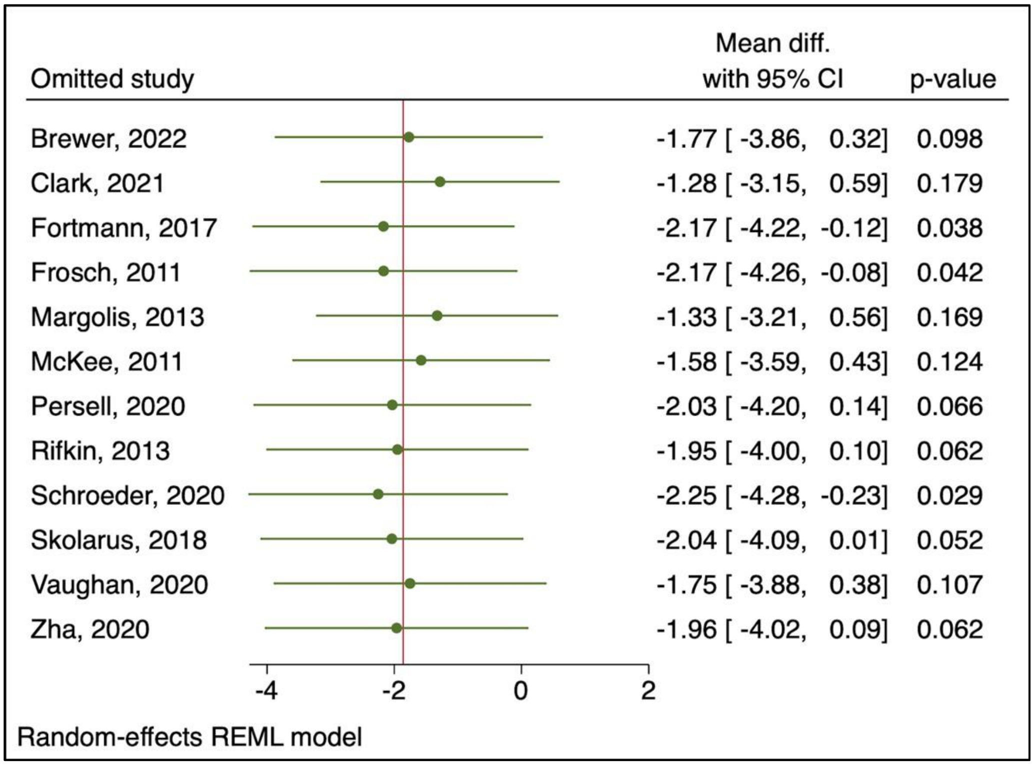

© 2024 Katz ME et al. *JAMA Network Open*.

eFigure 7. Risk of bias assessment for included studies.

Study	D1	D2	D3	D4	D5	Overall
Anderson 2010	+	+	+	+	+	+
Bennett 2018	+	+	+	+	+	+
Bennett 2012	+	+	+	+	+	+
Bosworth 2018	+	+	+	+	+	+
Bosworth 2009	+	+	+	+	+	+
Bove 2013	+	+	+	+	+	+
Brennan 2010	+	+	+	+	+	+
Brewer 2022	-	-	+	+	+	+
Chandler 2019	+	+	+	+	+	+
Clark 2021	-	-	+	+	+	+
Crowley 2013	+	+	+	-	-	-
Fortmann 2017	+	+	+	+	+	+
Frias 2017	-	-	+	+	+	+
Frosch 2011	+	+	+	+	+	+
Margolis 2013	+	+	+	+	+	+
McKee 2011	+	+	-	+	+	+
Morawski 2018	+	+	+	+	+	+
Persell 2020	+	+	+	+	+	+
Rifkin 2013	+	+	+	+	+	+
Still 2020	+	+	+	+	+	+
Tuot 2019	+	-	+	+	+	+
Vaughan 2020	+	+	+	+	+	+
Zha 2020	+	+	+	+	+	+
Skolarus 2018	+	+	-	+	+	+
Naqvi 2022	+	+	-	+	+	+
Schroeder 2020	+	+	+	+	+	+
Shea 2009	+	+	+	+	+	+

Risk of bias domains

Domains:
D1: Bias arising from the randomization process.
D2: Bias due to deviations from intended intervention.
D3: Bias due to missing outcome data.
D4: Bias in measurement of the outcome.
D5: Bias in selection of the reported result.

Judgement
- Some concerns
+ Low

© 2024 Katz ME et al. *JAMA Network Open*.
