# Supplementary material for: Digital Health Interventions for Hypertension Management in US Populations Experiencing Health Disparities: A Systematic Review and Meta-Analysis
Source: JAMA Netw Open. 2024 Feb 14;7(2):e2356070. doi: 10.1001/jamanetworkopen.2023.56070 (PMC10867699; doi:10.1001/jamanetworkopen.2023.56070)
Supplement: Supplement 2. — Data Sharing Statement [file jamanetwopen-e2356070-s002.pdf]

Data Sharing Statement

Katz. Digital Health Interventions for Hypertension Management in US Populations Experiencing Health Disparities. *JAMA Netw Open*. Published February 14, 2024. doi:10.1001/jamanetworkopen.2023.56070

Data
Data available: Yes
Data types: Data dictionary
How to access data: Upon request, we will share the literature review, data dictionary, and analytic code. Please contact: erica.spatz@yale.edu
When available: With publication

Supporting Documents
Document types: Statistical/analytic code
How to access documents: Please contact: erica.spatz@yale.edu
When available: With publication

Additional Information
Who can access the data: Anyone requesting the data.
Types of analyses: For any purpose
Mechanisms of data availability: With investigator support
Any additional restrictions: None
